# The Phenomenon of Self-Induced Diastereomeric Anisochrony and Its Implications in NMR Spectroscopy

**DOI:** 10.3390/molecules28196854

**Published:** 2023-09-28

**Authors:** Federica Aiello, Gloria Uccello Barretta, Federica Balzano, Fabio Spiaggia

**Affiliations:** 1 National Research Council, Institute for Chemical and Physical Processes (CNR-IPCF), Via G. Moruzzi 1, 56124 Pisa, Italy; 2 Department of Chemistry and Industrial Chemistry, University of Pisa, Via G. Moruzzi 13, 56124 Pisa, Italy; federica.balzano@unipi.it (F.B.); f.spiaggia@studenti.unipi.it (F.S.)

**Keywords:** self-aggregation, homodimers, heterodimers, nonequivalence, dimerization constant, enantiomers, chirality, SIDA, chiral analysis, NMR

## Abstract

Nuclear magnetic resonance (NMR) spectroscopy is an analytical technique largely applied in the analysis of discrimination processes involving enantiomeric substrates and chiral agents, which can interact with the analyte either via covalent bonding or via formation of diastereomeric solvates. However, enantiodiscrimination has been observed, in some cases, even in the absence of any additional chiral selector. The reasons behind this phenomenon must be found in the capability of some chiral substrates to interact with themselves by forming diastereomeric solvates in solution that can generate nonequivalences in the NMR spectra of enantiomerically enriched mixtures. As a result, differentiation of enantiomers is observed, thus allowing the quantification of the enantiomeric composition of the mixture under investigation. The tendency of certain substrates to self-aggregate and to generate diastereomeric adducts in solution can be defined as Self-Induced Diastereomeric Anisochrony (SIDA), but other acronyms have been used to refer to this phenomenon. In the present work, an overview of SIDA processes investigated via NMR spectroscopy will be provided, with a particular emphasis on the nature of the substrates involved, on the interaction mechanisms at the basis of the phenomenon, and on theoretical treatments proposed in the literature to explain them.

## 1. Introduction

Self-Induced Diastereomeric Anisochrony (SIDA) is a phenomenon that can be observed in the NMR analysis of chiral compounds, and it refers to the ability of enantiomeric substrates to self-associate in solution and form diastereomeric supramolecular aggregates that can be distinguished without the need to use a chiral auxiliary. This acronym was introduced by Ouryupin and coworkers [1]. Many other terms have been used to describe this phenomenon within the framework of NMR spectroscopy, starting from the “solute-solute interaction of enantiomers”, which was the term proposed by Williams et al. in 1969, when the phenomenon was first described [2]. The acronyms SCAD (Statistically Controlled Associate Diastereomerism) and SCADA (Statistically Controlled Anisochronism of Diastereomeric Association) were employed by Kabachnik et al. [3,4] for describing the self-aggregation of enantiomers. In the same period, Harger used the term “NMR nonequivalence of enantiomers” [5]. “Self-induced nonequivalence”, “self-discrimination of enantiomers”, and “Self-Induced Anisochrony” (SIA) were employed by Dobashi et al. [6], Luchinat and Roelens [7], and Giordano et al. [8], respectively. The last acronym that to our knowledge is employed in the literature is SIRE, which stands for “Self-Induced Recognition of Enantiomers”, introduced by Szántay and Demeter [9]. 

Different terms can be found in the literature in relationship to other analytical techniques [10]. As an example, the acronym “Self-Disproportionation of Enantiomers” (SDE), related to chromatography, was introduced by Soloshonok [11] and later was applied also to other physicochemical processes, such as sublimation, crystallization, and ultracentrifugation [12].

**Figure 1:**
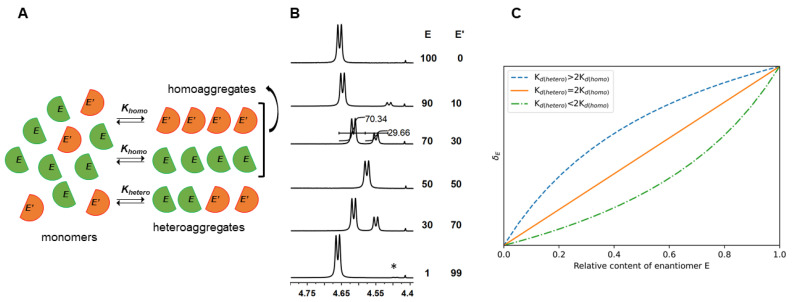
(**A**) formation of diastereomeric homo- and heteroaggregates starting from monomeric species. (**B**) splitting of signals in enantiomerically enriched samples; * indicates the signal of the minor enantiomer. (**C**) chemical shift vs. enantiomer composition profiles in the case of K_d(hetero)_ = 2K_d(homo)_ (solid line, orange), K_d(hetero)_ > 2K_d(homo)_ (dashed line, blue), and K_d(hetero)_ < 2K_d(homo)_ (dash-dotted line, green).

As briefly mentioned before, this phenomenon is related to the propensity of enantiomeric substrates (E and E′) to self-associate in solution, thus forming *homo*- or *hetero*aggregates (Figure 1).

The two supramolecular aggregates are in a diastereomeric relationship and, therefore, can be distinguished via NMR. When fast-exchange conditions (with respect to the NMR timescale) hold, the chemical shift of the resonances observed in the NMR spectra is a weighted average of the monomer and the *n*-mers present in solution in their homo- and heteroaggregate forms. In the condition in which only dimeric aggregates (homodimers, EE and E′E′, and heterodimers, EE′) are present, the chemical shifts observed for the E- (δ_E(obs)_) and E′-enantiomer (δ_E′(obs)_) are reported in Equations (1) and (2), respectively:

δ_E(obs)_ = x_E_ δ_m_ + x_EE_ δ_homo_ + x_EE′_^E^ δ_hetero_, (1)

δ_E′(obs)_ = x_E′_ δ_m_ + x_E′E′_ δ_homo_ + x_EE′_^E′^ δ_hetero_, (2)

where δ_m/homo/hetero_ are the chemical shifts of the monomer (E or E′), the homo- (EE or E′E′), and heterochiral dimers (EE′), and x_E_ = [E]/C_E_ is the fraction of the monomeric E-enantiomer with respect to C_E_ (molar concentration of the E-enantiomer), x_EE_ = 2[EE]/C_E_ is the fraction of the E-enantiomer in the EE homodimer, and x_EE′_^E^ = [EE′]/C_E_ is the fraction of the E-enantiomer in the EE′ heterodimer. Equal definitions apply for the E′-enantiomer. 

When analyzing an enantiopure substrate, only one set of signals is observed, given that the solute–solute interactions involve species with the same chirality. In a racemic mixture, instead, the interactions may occur between species with both the same chirality (formation of homoaggregates) and the opposite one (formation of heteroaggregates); also, in this case, one set of signals is generated, but their chemical shifts will differ from the enantiopure sample due to the contribution of the second interaction. Finally, in an enantiomerically enriched mixture, the most abundant enantiomer forms mainly homoaggregates, whereas the one in defect is involved mainly in heterochiral interactions. If, for instance, the E-enantiomer is the most abundant one in solution, the term x_EE_ δ_homo_ will prevail over x_EE′_^E^ δ_hetero_ (Equation (1)); on the contrary, for the E′-enantiomer, the term x_EE′_^E‘^ δ_hetero_ will be more significant than x_E′E′_ δ_homo_ (Equation (2)), hence δ_E(obs)_ ≠ δ_E′(obs)_. In this case, therefore, the splitting of signals is observed due to the different contributions to the aggregation processes for each enantiomer. The nonequivalence (Δδ), i.e., the chemical shift difference measured for the split signals of the two enantiomers, will depend on the enantiomeric excess (ee) of the mixture (Figure 1), and their integrated areas will reflect the enantiomeric composition of the sample analyzed.

As already observed in chiral discrimination phenomena involving the use of chiral auxiliaries [13], the magnitude of the nonequivalence can be affected by the following experimental conditions: (i) the choice of solvent (if the chiral discrimination is related to hydrogen-bond interactions, polar solvents will disfavor the process, whereas low polar ones will favor it); (ii) the total concentration of the solution (high concentrations will promote the aggregation); and (iii) the temperature (high temperature will increase the molecular tumbling and, hence, will favor the presence of monomeric species).

The recognition of this phenomenon, which is identified more clearly by using other analytical techniques (e.g., chromatography), is not so straightforward in NMR spectroscopy, mainly because of the relatively low concentrations used for the analysis that can disfavor the aggregation propensity of the substrates. For this reason, many NMR studies are carried out without taking into consideration the possible occurrence of such phenomenon and its implication in chiral analysis [14].

**Figure 2:**
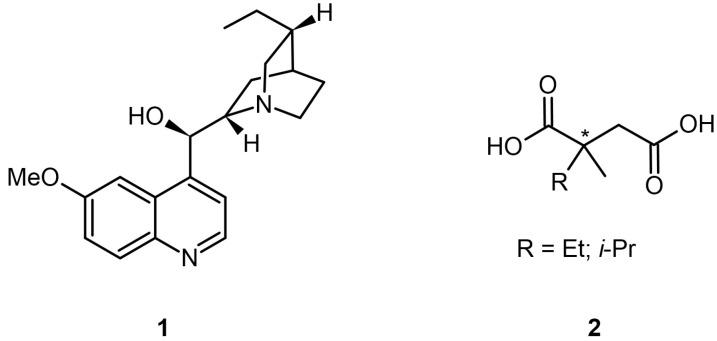
Dihydroquinine (**1**) and α-alkyl derivatives of α-methylsuccinic acid (**2**). * Indicates the chiral center of the substrate.

Dihydroquinine (**1**, Figure 2) was the first substrate for which the phenomenon was observed: the comparison of the NMR spectra recorded in CDCl_3_ for the enantiomerically pure and the racemic sample revealed, in fact, that the chemical shifts of the proton resonances were not coincident [2]. In addition, the proton spectra of enantiomerically enriched samples showed splitting of some signals, with the signals with minor intensity resonating at higher frequencies and the relative integrated areas corresponding to the relative enantiomers content, allowing the ee to be calculated. As expected, a dependence from the solvent was found, since when the spectra were recorded in deuterated methanol, the nonequivalence was less enhanced given that the polar solvent hampered the solute–solute aggregation, based on hydrogen-bond-like interactions. The occurrence of diastereomeric association processes in solution characterized by different stability was hypothesized a few years later, when differentiation between the spectra of enantiopure, racemic, and enriched mixtures was observed for α-alkyl derivatives of α-methylsuccinic acid (**2**, Figure 2) [15].

The present review focuses on the description of this phenomenon in NMR spectroscopy and on its exploitation as a tool for the determination of the enantiomeric purity of chiral substrates. Independently from the terms used in the literature, we adopt the acronym SIDA all over the review for sake of clarity. The first part aims at providing a deep theoretical treatment of the phenomenon by comparing the different approaches used over the years. A brief paragraph is dedicated to the methods available for the determination of the homo- and heterodimerization constants. The second part of the review covers an overview of selected NMR studies of chiral compounds, spanning from the earliest reports to some of the most recent ones, by focusing in particular on the findings that have been published in the last five years.

## 2. Theoretical Dissertation

In this section, the different theoretical treatments developed in the past years are discussed in chronological order. In the attempt to adopt a single formalism, as already found in the introduction, the general notation listed in Table 1 is used. 

One of the first theoretical treatments adopted for explaining the phenomenon of SIDA was proposed in 1976 by Kabachnik and coworkers [3]. They focused on the formation of dimeric species in solution and expressed the observed chemical shifts of generic E- and E′-enantiomers as a function of both concentration and the relative energy stability of the homo- and heterodimer (Equations (3) and (4)):

δ_E(obs)_ = (W_EE_ δ_homo_ + W_EE′_ δ_hetero_)/(W_EE_ + W_EE′_), (3)

δ_E′(obs)_ = (W_E′E′_ δ_homo_ + W_E′E_ δ_hetero_)/(W_E′E′_ + W_E′E_), (4)

where δ_homo_ (the chemical shift of the homodimers, EE or E′E′) and δ_hetero_ (the chemical shift of the heterodimer EE′) were termed by the authors as homo- and cross-shift, respectively; W_EE_ and W_EE′_ are statistically weighted contributions of the homo- and heteroaggregates to δ_E(obs)_. An equal definition applies to W_E′E′_ and W_E′E_ for the contribution to δ_E′(obs)_.

Equations (3) and (4) apply in the condition of fast exchange with respect to the NMR timescale (termed by Kabachnik and coworkers as the condition of Statistically Controlled Association). The statistical weights depend not only on the relative concentration of each enantiomer but also on the relative stabilities of the homo- and the heteroaggregates. As a result, the statistical weights allow to account for both analytical (i.e., the enantiomeric composition) and thermodynamic (i.e., preference toward formation of homo- or heterodimer) information. As an example, the statistical weight contribution for the homodimer (W_EE_) to δ_E(obs)_ can be written as α_homo_ *t*, where *t* is the relative content of the E-enantiomer, which can be defined as C_E_/(C_E_ + C_E′_), with C_E_ and C_E′_ being the molar concentrations of the E- and E′-enantiomer, respectively. α_homo_ is instead an Arrhenius-like parameter that accounts for the energy stability of the homodimer (Equation (5)):

α_homo_ = A_homo_ e^−Eh/(RT)^ (5)

where A_homo_ is a pre-exponential factor reminiscent of the same factor in the Arrhenius equation, E_h_ is the relative energy stability of the homodimer, R is the universal gas constant, and T is the absolute temperature. Analogously, based on the stability parameters for the heterodimer (α_hetero_), it is possible to write the statistical weight contribution for the heterodimer (W_EE′_) to δ_E(obs)_ as α_hetero_ (1 − *t*).

Identical equations can be written for the statistical weight contributions for both the homodimer and the heterodimer with respect to δ_E′(obs)_ (W_E′E′_ and W_E′E_). The ratio of the stability parameters α_hetero_/α_homo_ yields the stereospecificity of association of the enantiomers. The NMR spectrum is unable to give values of such parameters, and only their ratio can be provided. Substituting the statistical weight contributions and the ratio of the stability parameters into Equations (3) and (4) yields Equation (6) for the E-enantiomer and Equation (7) for the E′-enantiomer, respectively:

δ_E(obs)_ = (*t* δ_homo_ + (α_hetero_/α_homo_) (1 − *t*) δ_hetero_)/(*t* + (α_hetero_/α_homo_) (1 − *t*)), (6)

δ_E′(obs)_ = ((1 − *t*) δ_homo_ + (α_hetero_/α_homo_) *t* δ_hetero_)/((1 − *t*) + (α_hetero_/α_homo_) *t*). (7)

When α_hetero_/α_homo_ = 1, no stereospecificity is observed in the formation of the dimers, and δ_E(obs)_ and δ_E′(obs)_ show a linear dependency with respect to the relative content of E and E′ enantiomers. When the ratio is different from one, a departure from linearity can be observed in the plot of δ_E(obs)_ vs. *t*, and the curvature depends on the ratio α_hetero_/α_homo_. The authors deepened this theoretical treatment in their review [4], where the ratio between the stability parameters was denoted as m as in Equations (8) and (9).

δ_E(obs)_ = (*t* δ_homo_ + m (1 − *t*) δ_hetero_)/(*t* + m (1 − *t*)), (8)

δ_E′(obs)_ = ((1 − *t*) δ_homo_ + m *t* δ_hetero_)/((1 − *t*) + m *t*) (9)

In the case of a racemic mixture (*t* = 0.5), the two equations for the chemical shifts coincide (Equation (10)):

δ_E(obs)_ = δ_E′(obs)_ = (δ_homo_ + m δ_hetero_)/(1 + m), (10)

namely, the chemical shifts associated with the E- and E′-enantiomers coalesce into just one signal when a racemic mixture is prepared.

The fact that the chemical shifts measured in the racemic mixture are shifted with respect to the enantiomerically pure sample can be explained by considering, as mentioned above, that in the enantiomerically pure sample there is only the contribution of the homodimer, whereas in the racemic sample, the contribution of the heterodimer is also present. Kabachnik and coauthors referred to this phenomenon as *deviation*, which is a characteristic feature of SIDA [4]. They also extended their description to species which are not enantiomers but rather diastereomers (D/D′) [3,4]. In this case, if D and D′ are diastereomeric species capable of self-association, their relative chemical shifts can be expressed as Equations (11) and (12):

δ_D_ = (α_D_^D^ *t* δ_D_^D^ + α_D_^D′^ (1 − *t*) δ_D_^D′^)/(α_D_^D^ *t* + α_D_^D′^ (1 − *t*)), (11)

δ_D′_ = (α_D′_^D′^ (1 − *t*) δ_D′_^D′^ + α_D′_^D^ *t* δ_D′_^D^)/(α_D′_^D′^ (1 − *t*) + α_D′_^D^ *t*), (12)

α_D_^D^ and α_D′_^D′^ are the stability parameters that account for the formation of the DD and D′D′ homodimers, and α_D_^D′^ and α_D′_^D^ account for the formation of the heteroaggregates. If a mixture of self-aggregating D and D′ diastereomers is prepared, the terms α_D_^D′^ and α_D′_^D^ describe the stability of the D + D′ and D′ + D pairs, respectively; since they are essentially equal, the two stability parameters are also equal. The terms δ_D_^D′^ and δ_D′_^D^ are instead unequal and represent the shift of the nucleus of the D diastereomer when it aggregates with the D′ diastereomer and the shift of the nucleus of the D′ diastereomer when it aggregates with the D diastereomer, respectively. δ_D_^D^ and δ_D′_^D′^ are different between themselves and represent the chemical shift of the nucleus of the D diastereomer when it associates with itself and the shift of the D′ diastereomer when it associates with itself, respectively. When a relative content of D (*t*) equal to 0.5 is fed into the above equations, two different shifts are obtained, as expected in the case of a mixture containing two diastereomers.

Kabachnik and coworkers further extended this theoretical treatment in the case of a mixture containing N different stereoisomers. Specifically, the chemical shift of the *i*th component that self-associates with a general *j*th component can be expressed as shown in Equation (13):

(13) $$\delta_{i}=\sum_{i=1}^{N}W_{i}^{j}\delta_{i}^{j}/\sum_{j=1}^{N}W_{i}^{j}$$

where the statistical contribution $W_{i}^{j}$ is a function of the stability parameter *α_i_^j^* and of the concentration of the *i*th component.

A typical SIDA graph δ_E(obs)_ vs. *t* [4] cannot have minimum/maximum or any inflection points. Deviations to butterfly-type curves were observed for enantiomeric salts formed by chiral cations and chiral anions, due to the contribution of ionic pairing and hydrogen-bonded interactions. A deep description of these particular cases is provided in the literature [3,4]. 

The work published in 1977 by Kabachnik [16] can serve as an excellent guideline for the determination of the parameters introduced by the authors (m and δ_hetero_) for a mixture of enantiomers. Starting from Equation (8), by imposing the chemical shift associated with enantiomerically pure mixture (δ_homo_) to be zero, by expressing δ_E(obs)_ as a shift from δ_homo_, and linearizing, it is possible to express the chemical shift of E-enantiomer as reported in Equation (14).

1/δ_E(obs)_ = 1/δ_hetero_ + *t*/((1 − *t*) m δ_hetero_) (14)

By applying a linear regression model using *t*/(1 − *t*) as the independent variable, it would then be possible to determine δ_hetero_ and m from the intercept and the slope of the linear equation. Applying the same arguments for the chemical shift associated with the E′-enantiomer leads to Equation (15).

1/δ_E′(obs)_ = 1/δ_hetero_ + (1 − *t*)/(*t* m δ_hetero_) (15)

The energy difference between the homoaggregates and the hetero (or *cross*) aggregates can be determined from the definition of the stability parameters (Equation (5)). Calculating the ratio α_hetero_/α_homo_ and expressing this ratio as logarithms leads to Equation (16):

ln(α_hetero_/α_homo_) = ln(A_hetero_/A_homo_) + ΔE/(RT), (16)

with ΔE = E_homo_ − E_hetero_. Therefore, by measuring the ratio of the stability parameters at different temperatures and by plotting ln(α_hetero_/α_homo_) against 1/T, it is possible to determine ΔE.

**Figure 3:**
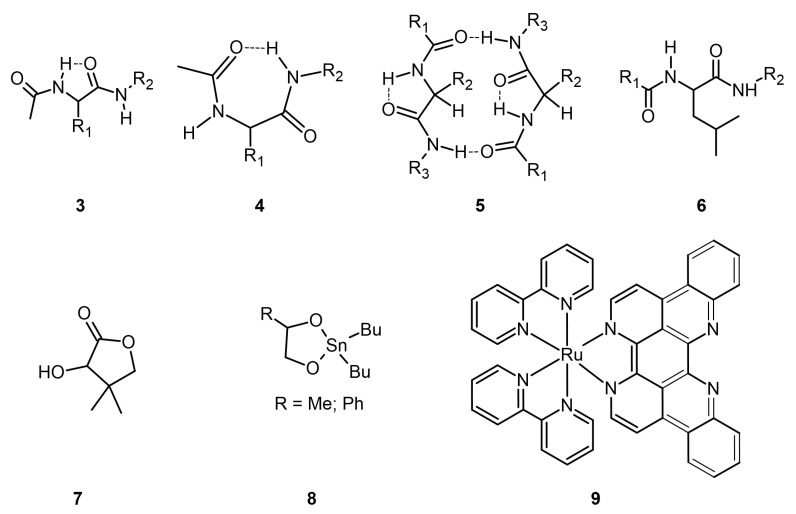
C_5_ (**3**) and C_7_ (**4**) conformations (R_1_, R_2_ = H, alkyl, CH_2_CH_2_SCH_3_, CH_2_Ar), isotactic dimer (**5**) (R_1_, R_2_, R_3_ = alkyl), general structure of leucine-like molecules (**6**) (R_1_, R_2_ = alkyl), pantolactone (**7**), dioxastannolanes (**8**), and example of octahedral chiral complex (**9**).

The main drawback of this theoretical treatment is that only dimeric species are considered in solution and no space is left for the existence of free monomeric species. In 1977, Cung et al. [17] first proposed a theoretical treatment in which monomeric isolated species of enantiomers are considered by thoroughly investigating the SIDA phenomenon in amino acid derivatives (see Section 3.2). The conformation of single molecules in diluted solutions had been studied few years earlier [18], and based on those results, it was concluded that the investigated *N*-acetyl amino acid amide systems can adopt two preferential conformations (C_5_ and C_7_, indicated as **3** and **4** in Figure 3) which are involved in an interconversion equilibrium. The interconversion equilibrium constant *k* between the two conformers can be defined as C_7_/C_5_.

It was demonstrated for a broad range of amino acid substrates that, as the concentration increased, the C_5_ conformers self-associated to form cyclic dimers. Specifically, if the two conformers have the same chiral configuration, they will form an isotactic dimer (EE or E′E′) (**5**, Figure 3), i.e., a dimer in which both the monomers have the same chiral configuration (a homodimer) (Equilibrium 1).

Equilibrium 1: 2E ⇆ EE and 2E′⇆E′E′

where E and E′ represent the monomers in the conformation capable of giving the dimeric adducts.

The equilibrium constant associated with Equilibrium 1 is given by Equation (17):

K_d(homo)_ = [EE]/[E]^2^ = [E′E′]/[E′]^2^ (17)

where [EE], [E′E′], [E], and [E′] represent the equilibrium concentrations of the homodimers and the monomers. Conversely, a syndiotactic or heterodimer can be formed when two monomers with opposite configurations self-associate (Equilibrium 2):

Equilibrium 2: E + E′ ⇆ EE′

The relative equilibrium constant for Equilibrium 2 is given by Equation (18):

K_d(hetero)_ = [EE′]/([E][E′]) (18)

where [EE′] is the equilibrium concentration of the heterodimer.

The mass balance equations for the E- and E′-enantiomers can be expressed in function of x_E_ and x_E′_ by using the equilibrium constants *k*, K_d(homo)_ and K_d(hetero)_, as shown in Equations (19) and (20) (a detailed treatment is provided in the literature [17]).

1 = x_E_ + x_EE_ + x_EE′_^E^ = x_E_ + K_1_ *t* C x_E_^2^ + K_2_ (1 − *t*) C x_E_ x_E′_, (19)

1 = x_E′_+ x_E′E′_ + x_EE′_^E′^ = x_E′_ + K_1_ (1 − *t*) C x_E′_^2^ + K_2_ *t* C x_E_ x_E′_ (20)

The constants K_1_ and K_2_ are dimerization equilibrium constants that account for the interconversion equilibrium between C_5_ and C_7_ and can be defined as reported in Equations (21) and (22).

K_1_ = 2K_d(homo)_/(1 + *k*)^2^, (21)

K_2_ = K_d(hetero)_/(1 + *k*)^2^ (22)

The ratio K_2_/K_1_ is a measure of the stereospecificity of the interacting monomers, similarly to what previously said about the stability parameter m. When this ratio is greater than one, a preference toward formation of the heterodimer is observed, whereas a ratio lower than one indicates preference toward formation of the homodimer. When the ratio equals one, there is no stereoselectivity in the association, i.e., no preferential dimerization toward the homodimer or the heterodimer. Another important point is that when K_1_ = K_2_, it also follows that x_E_ and x_E′_ are equal and independent from *t* provided that the total concentration C is maintained constant. Furthermore, the chemical shifts associated with the two enantiomers vary linearly with respect to *t*, as described in the lines that follow. 

With respect to Equations (1) and (2), in this particular case, where the monomeric species are involved in conformational equilibria,δ_m_ was then defined by the authors as the weighted average of the chemical shifts of the C_5_ (δ^C5^) and C_7_ (δ^C7^) conformers, according to Equation (23):

δ_m_ = (δ^C5^ + *k* δ^C7^)/(1 + *k*), (23)

where *k* is the interconversion equilibrium constant between C_5_ and C_7_. By expressing the fractions of the dimeric species in function of the equilibrium constants (Equations (21) and (22)), Equations (1) and (2) can be rewritten as Equations (24) and (25).

δ_E_ = δ_m_ x_E_ + δ_homo_ K_1_ *t* C x_E_^2^ + δ_hetero_ K_2_ (1 − *t*) C x_E_ x_E′_ (24)

δ_E′_ = δ_m_ x_E′_ + δ_homo_ K_1_ (1 − *t*) C x_E′_^2^ + δ_hetero_ K_2_ *t* C x_E_ x_E′_ (25)

As it has been previously expressed, the dependence between the chemical shifts and the enantiomeric composition *t* can be linear or not, depending on whether stereospecificity is present. Equations (24) and (25) describe the general case in which stereospecificity may be present or not.

Now, if the fraction of the E-enantiomer in the heterodimer is expressed as shown in Equation (26), it is then possible to express the chemical shift associated with the E-enantiomer as reported in Equation (27), which leads unequivocally to Equation (28):

x_EE′_^E^ = 1 − x_E_ − x_EE_ = 1 − x_E_ − K_1_ *t* C x_E_^2^, (26)

δ_E_ = δ_m_ x_E_ + δ_homo_ K_1_ *t* C x_E_^2^ + δ_hetero_ (1 − x_E_ − K_1_ *t* C x_E_^2^), (27)

δ_E_ = δ_hetero_ − (δ_hetero_ − δ_m_) x_E_ − (δ_hetero_ − δ_homo_) K_1_ *t* C x_E_^2^; (28)

Equation (28), when condition K_1_ = K_2_ (x_E′_ = x_E_ = cost) holds, can highlight the linearity between δ_E_ and *t* if the total concentration C is maintained constant. By applying the same argument for the E′-enantiomer, Equation (29) is obtained:

δ_E′_ = δ_hetero_ − (δ_hetero_ − δ_m_) x_E′_ − (δ_hetero_ − δ_homo_) K_1_ (1 − *t*) C x_E′_^2^, (29)

where δ_E′_ and *t* vary linearly with each other. It immediately follows that the nonequivalence (Δδ), namely the difference between the chemical shifts associated with the two enantiomers (δ_E_ − δ_E′_), has a linear dependence on the enantiomeric composition and, therefore, on the enantiomeric excess.

In a successive study published in 1978 [19], the authors investigated the self-discriminating property of leucine-like molecules (**6**, Figure 3). According to the authors’ analysis of IR, NMR, and vapor pressure data of the investigated molecules, the pure enantiomers tended to homoassociate to form higher-order aggregates instead of dimeric species. This behavior was responsible for an unexpected trend of the nonequivalence with respect to the total concentration, as it decreased as the total concentration increased. The authors provided a detailed theoretical treatment of this particular case, and interested readers are referred to [19] for more details. Briefly, they attributed the magnitude of the nonequivalence mainly to the presence of dimeric species; in the case of this amino acid derivative, the amount of such dimeric species was higher at lower total concentrations, i.e., their statistical weight was greater in more diluted solutions, hence the lower nonequivalences observed in concentrated solutions.

A similar theoretical treatment was proposed by Nakao and coworkers in their study on the dimerization and self-discrimination of pantolactone (**7**, Figure 3) [20]. Nonetheless, the authors were able to calculate the dimerization constant for the homodimer by using a different approach from the one proposed by Cung. They studied the relationship between the intensities of the OH and C=O IR stretching region and the concentration of enantiomerically pure l-pantolactone solutions. A method based on least squares devised by Sugeta [21] was then applied to determine the molar absorption coefficients, the association constant, and the monomer, homodimer, and heterodimer chemical shifts (as defined in Equations (1) and (2)). An important contribution from reference [20] is the way in which the absence of stereospecificity is derived. To be more precise, the homodimerization and heterodimerization equilibria for l- and d-pantolactone and the corresponding equilibrium constants can be defined similarly as above (see Equilibria (1) and (2) and Equations (17) and (18)).

The mass balance equations for generic E-and E′-enantiomers can be written according to Equations (30) and (31); summing each term of Equations (30) and (31) leads to Equation (32), expressed with respect to the total concentration C.

C_E_ = [E] + 2[EE] + [EE′], (30)

CE′ = [E′] + 2[E′E′] + [EE′], (31)

C = [E] + [E′] + 2[EE] + 2[E′E′] + 2[EE′] (32)

It is possible to define the total monomer equilibrium concentration as [M] = [E] + [E′]. The total dimer equilibrium concentration can be written as shown in Equation (33); expressing the equilibrium concentrations as a function of the equilibrium constants (Equations (17) and (18)) leads to Equation (34).

[dimers] = [EE] + [E′E′] + [EE′], (33)

[dimers] = [E]^2^ K_d(homo)_ + [E′]^2^ K_d(homo)_ + [E] [E′] K_d(hetero)_ (34)

In the case of a racemic solution, [E] = [E′] and, therefore, [E] = [E′] = ½ [M]. Substituting this condition in Equation (34) leads to Equation (35).

[dimers] = ½ [M]^2^ K_d(homo)_ + ¼ [M]^2^ K_d(hetero)_ = ¼ [M]^2^ (2K_d(homo)_ + K_d(hetero)_) (35)

When K_d(hetero)_ = 2K_d(homo)_, (i) the equilibrium concentration of all the dimeric species in a racemic mixture is the same as that of an enantiomerically pure solution (both samples being at the same analytical concentration), and (ii) no stereospecificity is observed in the formation of either the homodimer or the heterodimer. Condition (i) can be appreciated if K_d(hetero)_ = 2K_d(homo)_ is inserted into Equation (35) leading to Equation (36):

[dimers] = [M]^2^K_d(homo)_ → K_d(homo)_ = [dimers]/[M]^2^ (36)

Condition (ii) is the same as that found by Cung et al. (see Equations (21) and (22) when K_1_ = K_2_) [17]. In this case, the only source that allows SIDA to arise is the different chemical shifts of the homodimer and the heterodimer, and a linear relationship between the chemical shift of the E-enantiomer and *t* is observed. It is noteworthy that the theoretical description proposed by Nakao accounts for both the presence of monomeric and dimeric species in solution, as already presented by Cung [17].

The theoretical treatment proposed a year later by Luchinat and Roelens [7], instead, only accounts for the presence of dimeric species. In their work, the authors dealt with chiral dioxastannolanes (**8**, Figure 3) that, according to their evaluation of the literature data at the time, consisted of dimeric species only. In this case, the mass balance equations for the E- and E′-enantiomers become those reported in Equations (37) and (38):

C_E_ = 2 [EE] + [EE′], (37)

C_E′_ = 2 [E′E′] + [EE′] (38)

In the condition of fast exchange with respect to the NMR timescale, the chemical shifts of the E- and E′-enantiomers can be written as reported in Equations (39) and (40):

δ_E_ = 2 [EE] δ_homo_/C_E_ + [EE′] δ_hetero_/C_E_, (39)

δ_E′_ = 2 [E′E′] δ_homo_/C_E′_ + [EE′] δ_hetero_/C_E′_ (40)

If the equilibrium concentration of EE is defined as [EE] = x, it immediately follows that [EE′] = C_E_ − 2x. When [EE′] is substituted into Equation (38), [E′E′] can be expressed as shown in Equation (41):

[E′E′] = x + (C_E′_ − C_E_)/2 (41)

When the equilibrium concentrations are substituted into Equations (39) and (40), after some rearrangements, they can be rewritten as Equations (42) and (43).

δ_E_ = δ_hetero_ − 2 (δ_hetero_ − δ_homo_) x/C_E_, (42)

δ_E′_ = δ_homo_ + (C_E_/C_E′_ − 2x/C_E′_) (δ_hetero_ − δ_homo_) (43)

The nonequivalence (Δδ = δ_E_ − δ_E′_) can be expressed according to Equation (44).

Δδ = (δ_hetero_ − δ_homo_) (C_E_ − 2x) (1/C_E_ − 1/C_E′_) (44)

Now, if only dimeric species are present in solution, the only equilibrium to be considered is the interchange between dimers (Equilibrium 3):

Equilibrium 3: EE *+* E′E′ ⇆ 2EE′,

and the constant relative to the equilibrium is given in Equation (45).

K = [EE′]^2^/([EE][E′E′]) (45)

The value of K obtained from Equation (45) yields the stereospecificity of association between the dimers. When the equilibrium concentrations are substituted into Equation (45), Equation (46) is obtained. Rearranging with respect to x yields Equation (47).

K = (C_E_ − 2x)^2^/(x^2^ + x (C_E′_ − C_E_)/2), (46)

(K − 4) x^2^ + x [K (C_E′_ − C_E_) + 8 C_E_]/2 − C_E_^2^ = 0 (47)

When K = 4, no stereospecificity is present in the association of the dimers, and the entity of the nonequivalence, under conditions of fast exchange, depends only on the different stereochemical arrangements of the homodimer and the heterodimer. The equilibrium concentration of EE(x) can be easily calculated when K = 4 holds, according to Equation (48):

x = C_E_^2^/(2 (C_E_ + C_E′_)) = *t*C_E_/2, (48)

When Equation (48) is fed into Equation (44), and recalling that *t*/(1 − *t*) = C_E_/C_E′_, Equation (49) is obtained.

Δδ = (δ_hetero_ − δ_homo_) (1 − 2*t*) = (δ_hetero_ − δ_homo_) ee (49)

Equation (49) means that, when K = 4 holds, a linear relationship exists between the nonequivalence and the enantiomeric excess (ee). It immediately follows that values of K > 4 lead to preferentially form the heterodimer, while values of K < 4 lead to preferentially form the homodimers (see Equation (45)). Besides, when K > 4, the nonequivalence vs. ee curve behaves as a concave function, while, when K < 4, the curve behaves as a convex function. This means that the examination of the nonequivalence vs. ee curve can lead to qualitatively evaluate whether the homodimer or the heterodimer is the favored species. Another important conclusion is that, in the presence of dimers only, the nonequivalence is independent from the total concentration of the investigated mixtures. The “K = 4” condition is essentially identical to the “m = 1” condition seen above in Kabachnik’s treatment [4], namely indicating a total absence of stereoselectivity in the aggregation processes of enantiomeric substrates.

In 1995, Fedin and Davankov [22] unified Luchinat and Roelens’ theoretical treatment with the one proposed by Kabachnik et al. Specifically, the authors defined Equilibrium (3) in its inverse form. Therefore, according to this definition, K = [EE] [E′E′]/[EE′]^2^. By defining m as α_homo_/α_hetero_, the authors correlated K and m as reported in Equation (50).

K = m^2^/4 (50)

In this way, the authors could demonstrate that both m = 1 and K = 1/4 equally imply an absence of stereospecificity in the aggregation process.

One of the last theoretical treatments proposed for SIDA is the one by Gut et al. in 2002 [23]. The authors investigated the stereoselective dimerization of octahedral chiral metal complexes (**9**, Figure 3). Their treatment is somewhat superimposable to that proposed by Nakao et al., as it accounts for the presence of monomeric species in solution. Additionally, it provides a method for calculating the heterodimer formation constant. Indeed, Equation (35) derived from the treatment by Nakao et al. is essentially the same as that derived by Gut and coworkers (Equation (51)):

[dimers]/[M]^2^ = (2 K_d(homo)_ + K_d(hetero)_)/4 = K_mean_ (51)

The term (2 K_d(homo)_ + K_d(hetero)_)/4 only contains constants and, therefore, its result must be a constant, which is termed by the authors as a “mean” dimerization constant. This constant accounts for the formation of homo- and heterodimeric species in racemic mixtures and can be used to determine the heterodimer formation constant.

The last theoretical description that can be presented is the one proposed by Szakács et al. in their review [24]. For a mixture that contains the E- and E′-enantiomers of a chiral specimen, where molar concentrations are indicated as C_E_ and C_E′_ (total molar concentration C = C_E_ + C_E′_), Szakács et al.’s approach to a theoretical description of the SIDA phenomenon is based upon the following assumptions: (i) the enantiomers undergo self-association to give dimers only, (ii) homo- and heterodimers are formed only via a single geometrical arrangement, i.e., only one type of homo- and heterodimer are present in solution, and (iii) monomers and dimers undergo fast interchange with respect to the NMR timescale. The chemical shifts of the species that contain the E- and E′-enantiomers are the concentration-weighted average of the chemical shift of the interchanging species (Equations (1) and (2)). Also, the mass balance equations for both enantiomers (Equations (30) and (31)) and the formation equilibria of both homodimers and heterodimers (equilibria (1) and (2)) are the same as those previously defined. 

An additional equilibrium that is considered by the authors (on the basis of refs. [7,25]) is the interchange (Equilibrium (3)) between the dimeric species. From Equilibrium (3), the associated equilibrium constant (Equation (52) can be defined and expressed in function of Equations (17) and (18)):

K = [EE′]^2^/([EE][E′E′]) = (K_d(hetero)_/K_d(homo)_)^2^ (52)

Expressing the homodimer constant with respect to x_E_ and x_EE_ or x_E′_ and x_E′E′_ yields Equation (53):

K_d(homo)_ = x_EE_/(2x_E_^2^ *t* C) = x_E′E′_/(2x_E′_^2^ (1 − *t*) C) (53)

The heterodimer formation constant can be calculated as in Equations (54) and (55):

K_d(hetero)_ = x_EE′_^E^/(x_E_ x_E′_ C_E′_) = x_EE′_^E^/(x_E_ x_E′_ (1 − *t*) C) (54)

K_d(hetero)_ = x_EE′_^E′^/(x_E_ x_E′_ C_E_) = x_EE′_^E′^/(x_E_ x_E′_ *t* C) (55)

When the molar fractions of the dimers, obtained from Equations (53)–(55), are inserted into Equations (1) and (2), it possible to obtain Equations (56) and (57):

δ_E_ = x_E_ δ_m_ + 2K_d(homo)_ x_E_^2^ *t* C δ_homo_ + K_d(hetero)_ x_E_ x_E′_ (1 − *t*) C δ_hetero_ (56)

δ_E′_ = x_E′_ δ_m_ + 2K_d(homo)_ x_E′_^2^ (1 − *t*) C δ_homo_ + K_d(hetero)_ x_E_ x_E′_ *t* C δ_hetero_ (57)

The proposed mathematical treatment becomes simpler in the condition in which stereoselectivity is absent for dimer association (Equation (58)):

K_d(homo)_ = 2K_d(hetero)_ → K = (K_d(hetero)_/K_d(homo)_)^2^ = 4 (58)

At this point, the fraction of the E-enantiomer in the heterodimer can be expressed (Equation (59)) in the same way as by Cung and coworkers [17] in their theoretical description:

x_EE′_^E^ = 1 − x_E_ − 2K_d(homo)_ x_E_^2^ *t* C (59)

Inserting Equation (59) into Equation (1) leads to Equation (60):

δ_E_ = δ_hetero_ + x_E_ (δ_m_ − δ_hetero_) + 2K_d(homo)_ x_E_^2^ *t* C (δ_homo_ − δ_hetero_) (60)

A specular equation arises for δ_E′_ when the fraction of the E′-enantiomer in the heterodimer is defined similarly to Equation (59) and inserted into Equation (2). By applying some minor rearrangements, the nonequivalence (Δδ = δ_E_ − δ_E′_) can be expressed as in Equation (61):

Δδ = 2K_d(homo)_ C [*t* x_E_^2^ − (1 − *t*) x_E_^2^] (δ_homo_ − δ_hetero_) (61)

Reminding that x_E_ is constant and equal to x_E′_ in the condition K_d(hetero)_ = 2K_d(homo)_ at constant C, Equation (61) is linear with respect to *t* and, consequently, to the ee.

Two additional important points that arise from the Szakács et al. treatment are that (i) Equation (61) expresses that increasing the total concentration C leads to an increase in the nonequivalence Δδ in the case in which only dimeric species are present together with monomeric ones, but no higher-order aggregates are present, and that (ii) the condition of absence of stereoselectivity in aggregation processes (i.e., K = 4) is a common feature to both cases in which monomers and dimers coexist in the mixture and only dimers are present.

Finally, a specific phenomenon is the one indicated as atypical SIDA (aSIDA) [26,27]. When aSIDA occurs, no splitting of signals is observed in enantiomerically enriched samples but only a migration of resonances linked to variation in the enantiomeric composition. Possible causes of this particular outcome include low values of the dimerization constants and, hence, low quantities of dimers or/and low differences between the chemical shifts of the homodimer (δ_homo_) and the heterodimer (δ_hetero_) [24,28]. For unequivocally attributing the migration observed to the occurrence of aSIDA, NMR experiments at low temperatures should be performed, as lowering the temperature can increase the fraction of the dimeric aggregates and, therefore, the nonequivalence [24]. Additionally, low temperatures can also affect the stereochemical arrangements of the dimeric aggregates, thus influencing the chemical shifts of the homodimer and the heterodimer.

### 2.1. Calculation of Homodimer and Heterodimer Constants

Experimentally, the determination of the homodimer constant involves the building up of a “dilution curve”: the NMR spectra of enantiomerically pure samples at different concentrations are recorded, and the chemical shifts of the nucleus/nuclei involved in the association are measured. The obtained data are then treated to determine the dimerization constant along with the limiting monomer and homodimer chemical shifts. Some of the methods include iterative procedures [29,30] or graphical methods [31]. The analysis tools provided by the website Supramolecular.org (http://supramolecular.org [32], accessed on 10 May 2023) allow to determine the dimerization constants by employing both cooperative and non-cooperative dimerization models. For both models, it is possible to calculate the dimerization constant for the homodimer using two methods: the Nelder–Mead method, which is a simplex-based function minimization procedure [33], or the Limited-Memory BFGS (Broyden–Fletcher–Goldfarb–Shanno) optimization algorithm. It is also possible to determine the dimerization constant for the homodimer by writing simple scripts on Python or R and then applying nonlinear least squares to determine the unknown parameters. The function to be used for applying the nonlinear least squares is reported in Equation (62):

δ_obs_ = δ_m_ + (δ_homo_ − δ_m_) (√(1 + 8 K_d(homo)_ C) − 1)/(√(1 + 8 K_d(homo)_ C) + 1), (62)

where C is the total (i.e., analytical) concentration of one of the mixtures employed for the “dilution curve” and δ_obs_ is the chemical shift of the nucleus involved in the aggregation process. δ_m_, δ_homo_, and K_d(homo)_ are the monomer chemical shift, the homodimer chemical shift, and the dimerization constants, respectively, and represent the unknown parameters to be determined via nonlinear least squares. Derivation of Equation (62) is described in detail in the literature [30].

In order to calculate the heterodimer formation constant, it is firstly necessary to calculate the “mean” dimerization constant defined in Equation (51). After building a dilution curve of a racemic sample and applying one of the calculation methods for the dimerization constant abovementioned, the heterodimer constant can then be calculated according to Equation (63):

K_d(hetero)_ = 4K_mean_ − 2 K_d(homo)_ (63)

It is important to bear in mind that if there is no stereospecificity in the aggregation process (i.e., the plot of the chemical shift of one enantiomer vs. the relative content of the same enantiomer is linear), it then follows that K_d(hetero)_ = 2K_d(homo)_ [20,24], and K_mean_ equals K_d(homo)_ (Equation (64)):

K_mean_ = (2K_d(homo)_ + 2K_d(homo)_)/4 = K_d(homo)_ (64)

In conclusion, even if a linear plot in the enantiomeric titration is observed, the dilution curve experiment on racemic mixtures can serve as an additional confirmation of the absence of stereospecificity.

## 3. Classes of Chiral Compounds Involved in SIDA

In the following paragraphs, the classes of compounds able to originate SIDA in solution are described by reporting them in chronological order, i.e., from the first discovered and investigated to the most recent ones.

### 3.1. P-Derivatives

**Figure 4:**
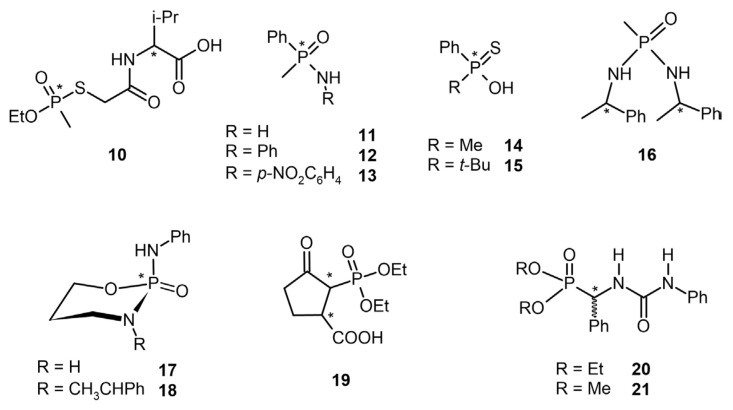
P-derivatives. * Indicates the chiral center of the substrates.

Organophosphorus substrates were among the first substrates for which splitting of signals was observed in the NMR analysis of enantiomerically enriched samples [3,16,34,35]. In the case of *N*-[(methylethoxyphosphinyl)thioglycolyl]valine (**10**, Figure 4) with two chiral centers (one on the phosphorus and one on one carbon), for which all four stereoisomers (*RR*, *SS*, *RS*, and *SR*) are obtainable in pure form, Kabachnik and coworkers found chemical shift differences in the ^31^P-{^1^H} NMR spectra of the individual stereoisomers and their mixtures [3,16]. For mixtures of unequal concentrations of enantiomers or stereoisomers, two to four (2^n^, n is the number of asymmetric centers) ^31^P NMR signals are observed; the relative intensities of which reflected the enantiomeric/diastereomeric mixture composition. In particular, the signal relative to the main enantiomer resonated at higher frequencies with respect to the minor one. To refer to this phenomenon, they first used the term “diastereomeric anisochrony” (DA), and rather than explaining it by considering the different stability of the adducts formed in solution, they related it to statistically controlled interactions, hence the term “statistically controlled associate diastereomerism” (SCAD, see Section 1). They focused on developing a statistical theory able to explain the experimental trend of DA depending on temperature and concentration as detected in mixtures of enantiomers and diastereomers.

Similar results were obtained by Harger in the ^1^H NMR analysis of alkylphenylphosphinic amides (**11**–**13**, Figure 4) bearing only one chiral center on the phosphorus [5,36,37]. The spectroscopic analysis revealed that the relative intensity of the split resonances is not dependent on the configuration of the enantiomer in excess, i.e., the chemical shift of the more intense resonance was the same whether the mixture is enriched in the *R*- or in the *S*-enantiomer, and it resonated at higher frequencies. This result indicated that the relative intensity is related to the association of the phosphinic amides in diastereomeric dimers formed by hydrogen bonded molecules with the same or opposite absolute configuration. A cyclic arrangement of the dimers was hypothesized on the basis of the chemical structure of the substrates involved. The same author proposed [37] optically active phosphinothioic acids (**14**, **15**, Figure 4), showing proton chemical shift difference for P-alkyl signals in racemic mixture with respect to the single enantiomer.

*N*,*N*′-bis(1-phenylethyl)diamidomethylphosphonates (**16**, Figure 4), obtained starting from an achiral methanephosphonic acid dichloride and chiral amines, were analyzed via ^31^P-{^1^H} NMR, with particular focus on the effect of the experimental conditions on the extent of the Self-Induced Diastereomeric Anisochrony [1]. It was observed that, for this substrate, the most favorable conditions for SIDA were achieved in toluene and at low temperatures (between −30 °C and −20 °C). The ^31^P NMR spectra of enantiomerically enriched mixtures showed that, also for this substrate, the most high-frequency shifted resonance belonged to the main enantiomer. The results observed, moreover, suggested that if the coupling reaction of the methanephosphonic acid with amines was quantitative, this approach could be proposed for the chiral analysis of amine involving their derivatization and, mainly, the tendency to self-discriminate of their corresponding derivatives.

1,3,2-Oxazaphosphorinanes **17** and **18** (Figure 4) were then chosen by the same authors as models for investigating the phenomenon of diastereomeric anisochrony in cyclic phosphoramidates and its dependence on various parameters [25]. The effect of temperature was analyzed both via ^1^H and via ^31^P-{^1^H} NMR spectroscopy, and a decrease up to −70 °C was associated to an improvement of the magnitude of nonequivalence in the ^31^P-{^1^H} NMR spectra of **17**, where the less abundant enantiomer resonated at higher frequencies with respect to the main one. The proton nuclei were more sensitive to the formation of the diastereomeric adducts, since higher nonequivalences were measured with equal temperatures compared with what observed for the phosphorus nuclei. Moreover, the difference between the chemical shifts measured for the enantiopure sample and for the racemic one (|δ_ep_ − δ_r_|) decreased with increasing temperature, becoming negligible at 40 °C for all the concentration range investigated. At temperatures equal to or lower than 20 °C, instead, an increase in the total concentration determined an increase in |δ_ep_ − δ_r_|. The derivatization of the cyclic nitrogen in **18** determined a lower number of functional groups possibly involved in hydrogen bond-like interactions, with a significant reduction in the splitting of signals in the scalemic mixtures. However, for this derivative, nonequivalence could be measured not only for the other amide resonance directly linked to the phosphorus but also for protons belonging to the derivatizing (α-methylbenzyl) group bound to the cyclic nitrogen, as for the methyl protons.

Racemic and optically active *trans*-3-carboxy-2-diethoxyphosphorylcyclopentanone **19** (Figure 4) were synthetized as precursors of racemic and optically active antibiotic sarkomycin, known for its antibacterial and antitumor activity. The analysis of the ^31^P-{^1^H} NMR spectra recorded for monitoring the progress of the resolution of this intermediate highlighted the occurrence of SIDA [38]. The formation of homo- and heterodimers was linked to the occurrence of intermolecular hydrogen bonds involving the carboxylic and the phosphoryl group of **19**, as confirmed by X-ray for the analogue crystalline compound, *trans*-3-carboxy-2-diphenylphosphinocyclopentanone.

More recently, Feringa investigated the self-discrimination processes with the aim to fine-tune the SIDA phenomenon as an analytical tool for NMR direct (i.e., without the need of a chiral auxiliary) determination of the enantiomeric purity of scalemic mixtures and as a convenient way to improve the screening of reaction conditions in asymmetric synthesis. Based on the literature data available on the classes of organic compounds capable to give SIDA, a series of α-ureidophosphonates, which was expected to generate diastereomeric adducts in solution, was synthesized and investigated via NMR and chromatography [39]. The ^1^H and ^31^P-{^1^H} NMR analysis of the crude derivative **20** (Figure 4) was performed in CDCl_3_ and splitting of signals was observed for the amide proton and the phosphorus resonance, respectively. In the proton spectrum, the signal at higher chemical shifts belonged to the main enantiomer, whereas the phosphorus resonances followed an opposite trend, i.e., the minor enantiomer resonated at higher frequencies. The integration of the split signals performed in both spectra confirmed the ee expected based on the enantiomeric purity of the synthetic precursors employed for the preparation of the substrate. Solvents with different polarities were then screened with the aim of identifying those that did not compete with the SIDA phenomenon. Unsurprisingly, no splitting was observed in highly polar solvents (DMSO-d_6_, CD_3_OD, DMF-d_7_). Similar nonequivalences were obtained in CD_2_Cl_2_ and toluene-d_8_ with respect to CDCl_3_, whereas a lower splitting was observed in the proton spectra recorded in acetonitrile-d_3_, where it was not possible to determine the ee of the sample by signals integration due to the absence of separation at the baseline. Interestingly, in the latter solvent the main enantiomer was centered at the lowest chemical shift for both proton and phosphorus nuclei. The trend of nonequivalences indicated that the aggregation phenomena were driven by the occurrence of intermolecular hydrogen bonds between the urea moieties and the phosphonate groups. DOSY (Diffusion Ordered SpectroscopY) experiments were performed on derivative **21** in different solvents to gain information on the molecular sizes of the aggregates and to confirm the formation of dimers. While the diffusion coefficient measured in CDCl_3_ was in agreement with the formation of dimeric species, the value measured in DMSO-d_6_ confirmed the main presence of monomers in solution, due to the polarity of the solvent that disfavored the aggregation, as already observed in the ^1^H NMR spectra. The homo- and heteroassociation constants, calculated by means of the dilution method, were indicative of a well-favored dimerization process (K_d(homo)_ = 3.3 ± 0.8 × 10^3^ M^−1^, K_d(hetero)_ = 8.3 ± 0.2 × 10^3^ M^−1^) in a broad range of concentrations. The comparison between the values calculated for K_d(homo)_ and K_d(hetero)_ highlighted a preference for the formation of heterodimers. 

### 3.2. Amino Acid Derivatives

**Figure 5:**
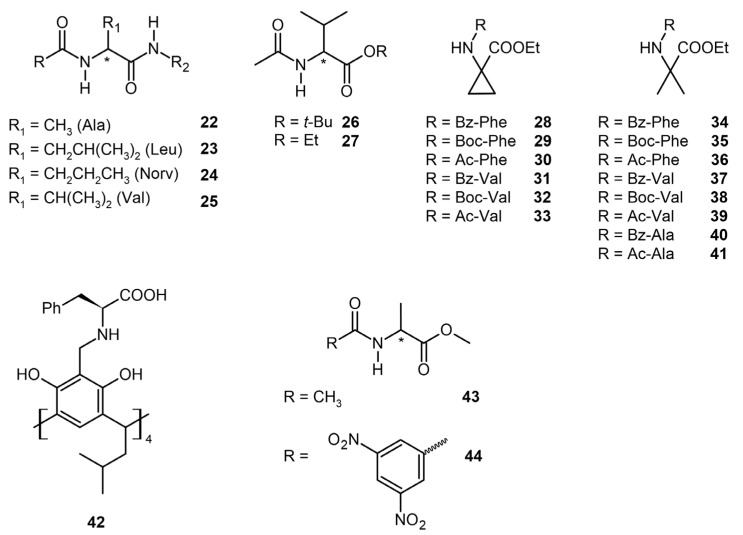
Amino acid derivatives. For **22**–**25**: R, R_2_ = alkyl groups. For **28**–**41**: Ac = acetyl; Boc = *tert*-butoxycarbonyl; Bz = benzoyl. * Indicates the chiral center of the substrates.

Diastereomeric association phenomena were observed in amino acid derivatives and were ascribed to the occurrence of hydrogen-bond interactions between the NH and the C=O groups of the substrates. Cung and coworkers focused on the analysis in CCl_4_ of derivatives of leucine, norvaline, alanine, and valine (**22**–**25**, Figure 5) [17,19,40,41]. For all the analyzed substrates, the splitting of signals due to SIDA resulted in the most abundant enantiomer resonating at lower chemical shifts with respect to the minor ones. l-leucine substrate was the only one involved in the formation of trimers or higher-order aggregates, while for the other l-derivatives, the intermolecular interactions mainly led to the formation of cyclic dimers. The comparison of the proton spectra recorded for a racemic mixture and for an enantiopure sample highlighted a different tendency to aggregate, more enhanced in the racemate, except for the least bulky alanine, where no stereoselectivity was observed. This result was explained by considering that in the homodimers the substituents of the amino acid chain are faced to the same part of the plane (see **5** in Figure 3), while in the heterodimer, they are faced on the opposite part, thus reducing the steric hindrance of the supramolecular aggregate. As a matter of fact, the stereochemical contribution is one of the discriminating factors that could drive toward the formation of a prevalent diastereomeric adduct.

Formation of homo- and heterochiral aggregates via intermolecular hydrogen bonds was observed also for the enantiomers of *N*-acetylvaline *tert*-butyl ester (**26**, Figure 5) [6], in particular for the doublet resonance belonging to the amide proton. The splitting of signals observed in a scalemic mixture pointed out an opposite trend with respect to substrates **22**–**25**, since, in this case, the most abundant enantiomer was the one with the more deshielded resonances. The nonequivalence significantly decreased by increasing the temperature from −20 °C to 20 °C and by lowering the enantiomeric excess of the mixture. The analysis of ^1^H and ^13^C NMR spectra recorded in CCl_4_ at different concentrations pointed out a variation in chemical shift not only for the NH proton but also for both the amide and the ester C=O, indicating that the hydrogen-bond interaction involved both functional groups. When the same experiments were performed on the ethyl derivative **27**, a smaller nonequivalence was detected in scalemic mixtures at the lowest temperature, thus confirming the primary role played by bulky substituents in favoring self-association phenomena.

Amino acid derivatives containing cyclopropyl or isobutyric moieties constitute a useful tool in peptide-based molecular architecture, since their rigid structure can be exploited for lending a specific conformation to the peptide skeleton and for determining the adoption of a particular secondary structure. The resulting supramolecular arrangement can significantly affect the final properties, such as the molecular and chiral recognition, the resistance to biodegradation, the bioavailability, and the pharmacokinetics. The NMR analysis of synthetic products **28**–**41** (Figure 5) [42] revealed that such derivatives can generate diastereomeric anisochrony, with the split signals having the same relative position observed for derivatives **22**–**25**. The integration of the split signals corresponded once again to the enantiomeric composition of the mixture. A preferential formation of heterochiral dimers over homochiral ones was ascertained based on the observed chemical shift of the amide protons, which were more deshielded in the racemic samples compared with the enantiopure ones, as a consequence of a stronger hydrogen-bond interaction. The variation in temperature affected the chemical shifts of the amide protons of the aminocyclopropane ring in a major extent compared with the amino acid moiety, indicating that this fragment was more involved in the intermolecular interactions leading to the dimeric aggregates. The comparison between three different amino acid fragments (Ala, Phe, Val) and between three different protecting groups at the *N*-terminal residue (Boc, Bz, Ac) highlighted the influence of the *N*-protecting group and of the bulkiness of the side chain. As a matter of fact, the highest nonequivalences were measured when valine was present in the molecule skeleton and when Bz was used as protecting group. When the cyclopropyl ring was replaced by a glycine fragment, negligible nonequivalences were measured for the split signals. These findings supported the importance of having residues with no preference for a C_5_ conformation at the *C*-terminus (**3**, Figure 3) for observing diastereomeric anisochrony. Finally, by using a protecting group lacking a carbonyl functionality at the *N*-terminus, such as 2-hydroxy-1-naphthaldehyde, no nonequivalence was observed in the NMR spectra, suggesting that the protecting group may be involved in hydrogen-bond interactions as well, significantly contributing to the formation of the dimers.

Amino acids can be used for the derivatization of achiral macrocycles to obtain synthetic capsules to be exploited for mimicking the active sites of proteins or for studying the effect of the cavity sizes in the design of molecular containers and nanoreactors. The derivatization of resorc[4]arene macrocycles with l-phenylalanine (**42**, Figure 5) was one of the first examples of non-covalent chiral capsule endowed with both polar and apolar functionalities [43]. The formation of dimeric species was confirmed by X-ray and NMR DOSY experiments, and the analysis of proton and carbon NMR spectra of the product revealed the high symmetry (*D*_4_) of the derivative in the solution since a single set of sharp signals was observed. Such symmetry was justified by considering the formation of homodimeric species; however, it was not expected to be the preferred one due to the occurrence of steric repulsion between the amino acid fragments of the monomers. To investigate the propensity of the system toward the formation of heterodimers in solution, the d-derivative was synthesized (starting from d-phenylalanine) and mixed with **42**. The proton spectra did not change if the sample was directly analyzed in CDCl_3_ but, if prior to the recording of the spectrum the sample was treated with methanol or subjected to dissolve–evaporate cycles, the chemical shift of some resonances changed as a consequence of the quantitative formation of the heterodimer. This result was interpreted as proof that the heterodimer was thermodynamically more stable than the homodimer and that there was a high kinetic stability in CDCl_3_, since the formation of heterodimers was a slow process.

Trapp and coworkers analyzed the SIDA effect on alanine derivatives, which are common products of enantioselective hydrogenation catalysis [44]. No SIDA was observed in the proton NMR spectra recorded in C_6_D_6_ for different scalemic mixtures of *N*-acetylalanine methyl ester **43** (Figure 5), with the exception of the *N*-acetyl methyl group. Nevertheless, the nonequivalence in this case was not sufficient for allowing the determination of the ee by signal integration. On the contrary, significant splitting of signals was observed, both in C_6_D_6_ and in CDCl_3_, in the scalemic mixtures of the *N*-3,5-dinitrobenzoyl methyl ester derivative **44** (Figure 5); in particular, splitting was observed for the amide and the acetyl protons, the ester and the amide carbons and the quaternary aromatic carbon directly linked to the C=O. This outcome pointed out the contribution of π–π stacking intermolecular interactions involving the 3,5-dinitrobenzoyl moiety in enhancing the formation of diastereomeric adducts in solution. It is worth noting that, for the proton resonances and for the ester carbonyl, the prevalent enantiomer resonated at higher frequencies, whereas the opposite trend was observed for the amide carbonyl and for the quaternary carbon.

### 3.3. Chiral Amides, Imides, and Alcohols

**Figure 6:**
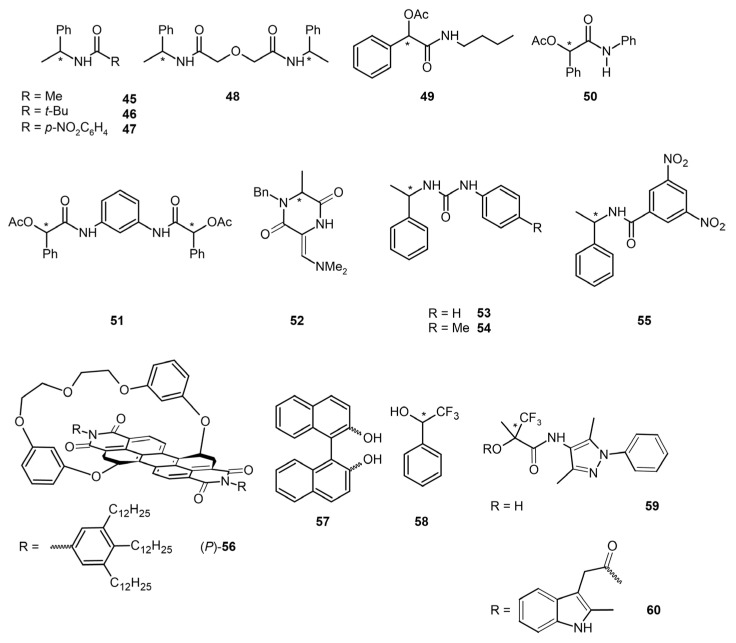
Chiral amides, imides, and alcohols. * Indicates the chiral center of the substrates.

Jursic and Goldberg analyzed a series of chiral carboxamides (**45**–**51**, Figure 6) that proved to give diastereomeric anisochrony [45]. The nonequivalences observed indicated a higher chemical shift for the minor enantiomer. Compared with alkylphenylphosphinic amides and the derivatives of norvaline, alanine and valine (discussed in the previous sections), for which the formation of cyclic dimers was hypothesized, in this case, the authors supposed the occurrence of linear diastereomeric interactions that could generate dimers, trimers and, more generally, *n*-mers.

By exploiting the chiral solvating properties of a diketopiperazine derivative toward chiral amide substrates, Wagger et al. found that a scalemic mixture of **52** (Figure 6) showed split signals in its ^1^H NMR spectrum recorded in CDCl_3_ solution [46].

A linear interaction was also hypothesized in the process leading to the formation of dimeric species for the urea derivatives of α-phenylethylamine **53** and **54** (Figure 6) [47]. Significant nonequivalences were observed for both the methyl and the amide protons in enantiomerically enriched samples, starting from a total concentration of 10 mM; as for compounds **45**–**51**, the prevailing enantiomer resonates at lower frequencies. Moreover, the large chemical shift variations observed for the amide protons of the urea moiety highlighted the main role played by intermolecular hydrogen bond interactions in the self-aggregation and suggested a double interaction involving the two NH protons of one monomer and the CO group of the other one. The occurrence of this double interaction was paramount for the SIDA, given that the corresponding amide derivatives of α-phenylethylamine were not involved in SIDA, except for compound **55** (Figure 6), endowed with two NO_2_ groups on the aromatic ring. The presence of two powerful electron-withdrawing groups likely enhanced its capability to be involved in hydrogen-bond interactions, even if with only one amide group.

π–π stacking was the main interaction that determined the formation of dimeric species in perylene bisimides, which constitute an interesting system due to their optical and electrochemical properties that make them useful as organic semiconductor materials. Safont-Sempere et al. investigated the self-assembly of the two enantiomers of **56** (Figure 6), by combining UV–vis, circular dichroism and NMR spectroscopy [48]. The UV–vis experiments revealed the formation of dimeric species in solution, in particular homodimers, as the homodimerization constant calculated was much higher than the heterodimeric one. The NMR analysis of enantiomerically enriched samples, performed in deuterated *n*-hexane, confirmed the formation of the two dimeric species, given that splitting of signals was observed for all perylene protons in enantiomerically enriched mixtures, with the most abundant enantiomer resonating at lower frequencies. Following these results, the investigation was extended to other chiral perylene bisimides endowed with oligoethylene glycol bridges of different lengths [49]: the NMR analysis performed at 58 °C allowed performing a quantitative analysis of the enantiomerically enriched samples to determine the ee of the mixtures by integration of the split proton signals.

**Figure 7:**
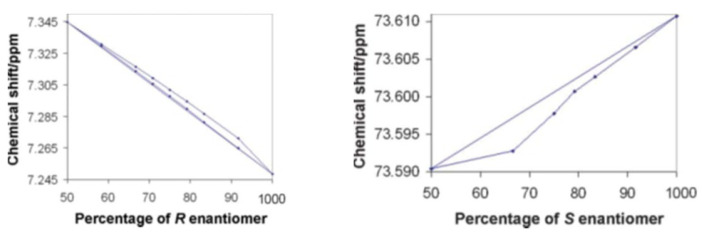
(**Left**) plot of δ_H_ vs. percentage *R*-enantiomer for **57**. (**Right**) plot of δ_C*_ vs. percentage *S*-enantiomer for **58**. Reprinted with permission from ref. [28].

Klika is one of the authors who analyzed the SIDA phenomenon in depth in the last fifteen years. He investigated the process mainly by exploiting chromatographic methods but also via NMR spectroscopy. One of the first NMR investigations was performed on 1,1′-bi-2-naphthol **57** and 1-phenyl-2,2,2-trifluoroethanol **58** (Figure 6) that had both shown self-disproportionation propensity in achiral-phase chromatography ([28] and references therein). The analysis performed on **57** in CDCl_3_ did not show any NMR splitting for the proton signals of the compound in scalemates, but rather shifting of resonances with respect to the racemic solution, which was linked to variations in the monomer–dimer equilibrium. The absence of splitting was explained by considering the amount of dimer formed in these conditions as insufficient (aSIDA). In deuterated benzene, a solvent with lower polarity compared with chloroform, splitting of resonances was instead observed for some protons in enantiomerically enriched samples, indicating a higher propensity to dimerization. The measurement of the diffusion coefficient of the species in the two solvents confirmed the greater extent of dimerization in benzene with respect to chloroform. By plotting the chemical shift variations vs. the percentage of *R*-enantiomer of **57** for the proton in α-position with respect to OH, the authors observed that for the major enantiomer a straight line was obtained, whereas a deviation from linearity was observed for the minor one (Figure 7, left). From this trend, it was inferred that the equilibrium between the homochiral and the heterochiral species is characterized by a small constant, and these findings were confirmed by calculations of the free energies.

Compound **58**, analyzed in *n*-hexane, behaved similarly to **57** in chloroform (i.e., no splitting but rather shift of signals). The chemical shift variation observed for its chiral carbon, when plotted against the percentage of *S*-enantiomer, revealed a distinct bend towards the δ_rac_ value (Figure 7, right). This result is an indication of a preferential formation of the heterodimer.

Recently, Klika analyzed the behavior of chiral alcohol **59** and its ester derivative **60** (Figure 6) [50]. In the attempt to analyze **59** in CDCl_3_, instantaneous precipitation was observed for the racemic solution, indicating that the formation of the heterochiral aggregates was favored in the solid state. This tendency forced the authors to analyze the substrate at very low concentrations (3 mg/mL), which disfavored the aggregation processes. As a matter of fact, the proton spectrum of the racemic mixture was nearly superimposable to that of the enantiopure sample, and no splitting of signal was observed in an enantiomerically enriched mixture (ee ~33%). Other deuterated solvents were tested, but either they were not able to solubilize the alcohol, or their polarity was not suitable for the aggregation. The absence of SIDA in polar solvents (acetone-d_6_, THF-d_8_, CD_3_CN) pointed at a process favored by hydrogen bond-based intermolecular interactions. The association in CDCl_3_ was confirmed by detecting the dipolar interaction by means of 1D-NOESY (Nuclear Overhauser Effect SpectroscopY) experiments performed on the enantiopure sample and on the racemic one at low concentration. The irradiation of the imidazolyl methyl protons caused, in both samples, enhancement of the signal belonging to the methyl group bound to the chiral carbon: such effect could be attributed only to intermolecular effect and confirmed the occurrence of self-association phenomena. However, the NOE effect was stronger in the racemic sample, suggesting the prevalence of heteroaggregation over homoaggregation. Accordingly, the analysis of the DOSY maps recorded for the two samples indicated a lower diffusion coefficient measured for the racemate in comparison with the enantiopure one, due to the stronger tendency to associate. The analysis of the ester derivative **60**, performed under the same experimental conditions, did not show any difference between the spectral profile of the enantiopure sample and the racemic one, which might indicate the absence of aggregation; however, chemical shift variations at different concentrations were still observed. These results were ascribed to weakened intermolecular hydrogen-bond interactions due to the derivatization of the hydroxyl group. The smaller SIDA effects observed suggested that intermolecular interactions were still based on hydrogen bonds but now involved the indolyl NH rather than the hydroxyl group. As a matter of fact, an increase in the deshielding of the indolyl NH proton was measured by increasing the sample concentration; splitting of the same proton signal was observed in scalemic samples as well. The analysis of the dipolar interactions confirmed the aggregation, but for this substrate, no substantial difference was observed between enantiopure sample and racemate, indicating that the homodimer and the heterodimer are equally favored. Such difference was linked to the increased flexibility of the ester derivative compared with alcohol, which determined a small difference between K_d(homo)_ and K_d(hetero)_. A different solution-state aggregation preference in comparison with **59** was inferred from the DOSY experiments, where a slightly lower diffusion coefficient was measured for the enantiopure sample; therefore, in this case, the formation of the homodimer was favored, even if to a small extent.

### 3.4. Chiral Drugs

**Figure 8:**
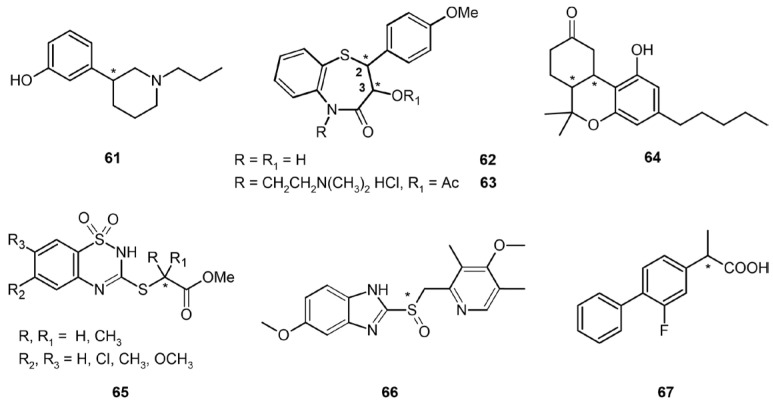
Chiral drugs. * Indicates the chiral center of the substrates.

3-(3-Hydroxyphenyl)-*N*-n-propylpiperidine (**61**, Figure 8) is a D2 dopamine receptor agonist that showed splitting of the signal belonging to its methyl group when an enantiomerically enriched mixture was analyzed in CDCl_3_. The two resonances were easily distinguished in the NMR spectrum of a 0.1 M sample; therefore, the enantiomeric purity of the synthesized substrate could be easily determined without the addition of any chiral auxiliaries [51].

Pantolactone (**7**, Figure 3) is the central intermediate in the synthesis of vitamin B5 ((*R*)-(+)-pantothenic acid) and dexpanthenol [52]. Given that only its *R*-stereoisomer has desirable biological activity, it is important to investigate its association mode in solution. The NMR investigation focused on the analysis of the chemical shifts measured for the hydroxy and methine resonances in the racemic sample, the enantiopure one, and in enantiomerically enriched mixtures to elucidate the self-association mechanism [20]. The occurrence of homo- and heteroassociation phenomena was confirmed by observing splitting of the signals belonging to such nuclei in the proton spectra recorded in CCl_4_; the chemical shifts spanned from 3.84 ppm and 4.03 ppm (pure enantiomer) to 3.40 ppm and 4.01 ppm (racemic sample) for the OH and the CH groups, respectively. For both resonances, the main enantiomer resonated at higher frequencies. The chemical shift of the hydroxyl group, which was the most sensitive to the variation in the enantiomeric composition of the mixture, was measured at different concentrations for the enantiomerically pure sample, and the formation of dimeric adducts was confirmed, since this aggregation mode better fitted the dilution curve. The same dilution experiments performed on the racemic sample and on the enantiomerically enriched ones revealed the absence of any selective association.

Enantiomerically pure 1,5-benzothiazepine (2*S*,3*S*)-**62** is one of the starting materials for the synthesis of diltiazem (2*S*,3*S*)-**63** (Figure 8), which is known for its calcium antagonist activity. The determination of the enantiomeric purity of this substrate, therefore, is paramount for the production of the drug. The NMR analysis of samples of enantiopure and racemic **62** revealed the occurrence of self-discrimination phenomena in CDCl_3_ solution [8], in particular, by observing the splitting of the proton resonance of the *p*-OCH_3_ group, for which the minor enantiomer resonated at higher frequencies. Nonequivalence was observed in very diluted solutions (till to 0.93 mM) of enantiomerically enriched samples. Also, in this case, self-discrimination was favored in halogenated and aromatic solvents, whereas the use of polar and/or strongly coordinating solvents (DMF-d_7_, DMSO-d_6_, MeOD-d_4_) hampered the interaction. The derivatization of the OH group in α-position with respect to the C=O functionality remarkably affected the nonequivalence, which was barely detected, while the replacement of the amide proton with a methyl group inhibited the formation of dimers, thus supporting the importance of intermolecular hydrogen bond-like interactions in the process. Interestingly, when changing the chiral configuration at C-3, the phenomenon was not observed.

Two enantiomers of the cannabinoid ketone **64** (Figure 8) were also able to interact in solution and to determine the formation of homo- and heteroaggregates in CDCl_3_ [53]. The OH resonance and the aromatic protons were shown to be the most sensitive to SIDA.

2-[(2H-1,2,4-Benzothiadiazine-1,1-dioxide-3-yl)thio]propanoic acids showed inhibitory activity towards aldose reductase, a key enzyme in the polyol pathway [54]. The NMR analysis of the corresponding methyl esters **65** (Figure 8) revealed that these substrates can interact in solution by forming diastereomeric adducts that can be distinguished spectroscopically [55]. Therefore, the enantiomeric composition of the mixtures could be determined without the use of any chiral auxiliaries, as confirmed by comparison with the data obtained by adding a chiral shift reagent to the samples. Nonequivalences were measured not only for the methoxy group but also for the amide and the aromatic resonances belonging to the substrates; while for the methoxy group and the amide proton the most shielded split signal belonged to the main enantiomer, the opposite trend was observed for the aromatic moiety.

Hentschel et al. employed a continuous-flow capillary microcoil for the ^1^H NMR monitoring of the enantiomeric titration of omeprazole (**66**, Figure 8) and highlighted the SIDA behavior of the drug in chloroform-d solution [56].

Flurbiprofen (**67**, Figure 8), a chiral drug belonging to the family of nonsteroidal anti-inflammatory drugs, was investigated by Klika and coworkers to evaluate its tendency to diastereomeric aggregation both in chromatography and in NMR spectroscopy, with the aim to correlate the data obtained from the two analytical approaches [57]. The final goal was indeed the rationalization and the prediction of chromatographic elution order starting from the NMR measurements. Moreover, the importance of investigating the occurrence of SIDA in this drug, currently available as racemate on the market, lies in the fact that the *R*-enantiomer has been investigated as a potential drug in a phase III clinical trial for Alzheimer’s disease. The NMR analysis was performed by measuring diffusion coefficients and relaxation times (T_1_ and T_2_). NMR measurements were carried out in four solvents (CDCl_3_, 1,4-dioxane-d_8_, acetonitrile-d_3_, and toluene-d_8_) and in one solvent mixture (cyclohexane-d_12_/methyl *tert*-butyl ether 4:1) at different concentrations. In this way, the authors investigated the solution–solute association preference and the SIDA magnitude used to identify the best one capable of favoring SIDA. Among those tested, toluene-d_8_ was the most performing one, as nonequivalences were detected in the ^1^H (methyl group), ^13^C (aromatic carbon bound to the chiral center), and ^19^F NMR spectra of enantiomerically enriched mixtures. The proton resonances of the main enantiomer were the most deshielded, whereas the most deshielded resonances in the ^13^C and ^19^F NMR spectra belonged to the minor antipode. In addition, differences in the diffusion coefficients between the enantiopure and the racemic sample were observed, and a prevalence for heterochiral association was pointed out. Weaker effects were obtained instead in CDCl_3_, with the favored formation of the homochiral species. The chromatographic results obtained do not allow one to find a clear correlation between the NMR and chromatographic data and, therefore, it was not possible to predict chromatographic outcomes based on the information obtained from the NMR analysis. This result was mainly attributed to the strong role played by the solvent polarity in favoring homo- or heterochiral association processes.

### 3.5. Natural Products

**Figure 9:**
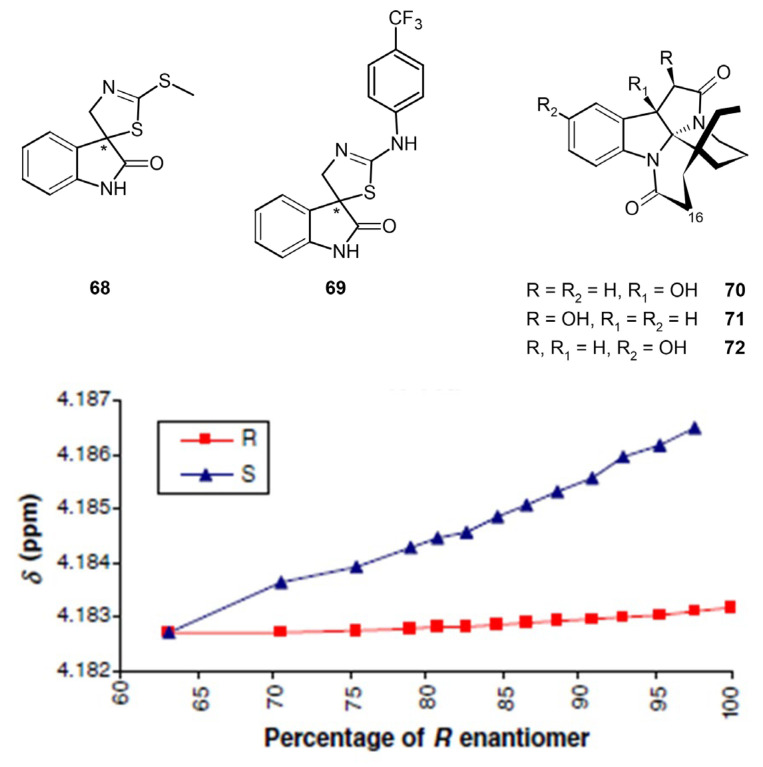
(**Top**) natural products. * Indicates the chiral center of the substrates. (**Bottom**) enantiomeric titration plot for **68**; δ vs. percentage of the *R*-enantiomer. Reprinted with permission from ref. [27].

Spirobrassinin (**68**, Figure 9) is a natural product belonging to the family of cruciferous phytoalexins, which are lipophilic substances with antimicrobial properties and anticancer activity. In 2010, Klika et al. analyzed this natural product by NMR in C_6_D_6_ [58] and noticed splitting of signals not only for the proton resonances (especially for the amide proton) but also for the other nuclei, in particular the carbonyl and the imine carbons, as well as the amide and the imine nitrogens, as a consequence of the formation of dimeric species. The conformation of the dimeric aggregates was investigated by means of computational modeling, and two complexation mechanisms were considered, linked to a bidentate amide-to-amide interaction (NH···OC) and to a monodentate amide-to-imine (NH···N) one, respectively. Overall, the homodimer was found to be more stable than the heterodimer, and the bidentate interaction was calculated to be the most energetically favored. However, the intermolecular dipolar effects detected in the NMR NOESY experiments revealed a contribution of both complexation mechanisms in the solution. The coexistence of the two complexes was explained by considering the occurrence of solute–solvent interactions, like π–π stacking, with the benzene molecules. An enantiomeric titration was then performed on compound **68** in attempt to confirm the favored formation of the homodimer [27]. Based on the chemical shift variation measured for the split signals of one of the protons in α-position with respect to the imide nitrogen (Figure 9), the authors inferred a preference for the formation of homochiral dimers in a C_6_D_6_ solution.

Some years later, SIDA phenomenon was also detected for a nonracemic solution of a 2′-amino analog of spirobrassinin **69** (Figure 9) in ^1^H and ^13^C NMR spectra recorded in a C_6_D_6_ solution [59].

Alkaloids scholarisine G (**70**) and leuconodines A (**71**) and C (**72**) (Figure 9) are natural products with different oxidation states. During their total synthesis, the NMR analysis of enantiomerically pure, enantiomerically enriched and racemic samples showed the occurrence of SIDA in CDCl_3_ [60]. The low-frequency shift observed for the proton H16 in **70**, belonging to the methylene in α-position with respect to the C=O group, was consistent with the crystalline heterodimeric structure; based on these data, the main formation of the heterodimer was considered. For the other two products, the SIDA was less pronounced and was confirmed by dilution experiments and X-ray analyses of the racemates and the pure enantiomers.

The occurrence of SIDA was observed also in the NMR spectra of synthetic myrtucommulones recorded in CDCl_3_. These compounds are acylphloroglucinol compounds that can be isolated from myrtle and other species of the *myrtaceae* family and are known for their anti-inflammatory and cytotoxic properties [61].

### 3.6. Metal Complexes

**Scheme 1:**
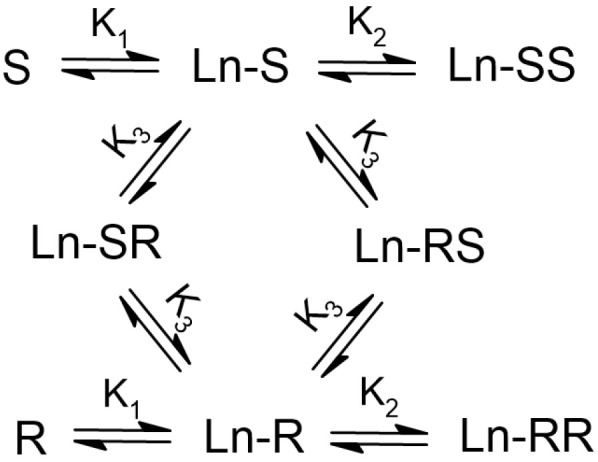
Equilibria involving the lanthanide agent and the chiral substrate.

Chiral lanthanide shift reagents (CLSRs) are, together with chiral solvating agents (CSAs), chiral auxiliaries that can interact with chiral substrates via noncovalent interactions, thus making it possible to differentiate among the two enantiomers [13]. However, there are some examples in the literature where enantiomeric differentiation was observed also by using an achiral lanthanide shift reagent. Nonequivalences were observed in the ^1^H NMR spectra for the methyl group of α-phenylethylamine and α-(2-thienyl)ethylamine in the presence of Eu(fod)_3_ and Eu(dpm)_3_ [62]. The nonequivalence depended not only on the enantiomeric excess of the sample but also on the concentration of the substrate and the shift reagent, with the highest value reached at amine/shift reagent molar ratio equal to 2:1. A lanthanide complex could act as an intermediate in the diastereomeric interaction, as explained with the equilibria reported in Scheme 1:

As a consequence of the different interactions that the lanthanide may establish in solution with the enantiomers of the substrate, four different complexes were present, Ln-*RR*, Ln-*SS*, Ln-*RS*, and Ln-*SR*, with the two couples in a diastereomeric relationship, hence generating resonances in the NMR spectra with different chemical shifts.

A similar approach to that of Ajisaka and Kainosho [62] was developed by Reuben in 1980 [63]. In this case, the addition of EuCl_3_ or PrCl_3_ in a 3:1 ligand/metal ratio to scalemic mixtures of lactate prompted the formation of the following metal complexes: Ml_3_, Md3, Ml2d, and Md2l, with the first one being in an enantiomeric relation with Md_3_ and in a diastereomeric one with Ml2d and Md2l. The different magnetic shielding of the three stereoisomers weighted on the relative distribution of the complexes was responsible for the differentiation of the enantiomers under condition of fast exchange. For this particular case, the author provided detailed phenomenological equations, and the interested reader is referred to the respective paper.

The propensity to give SIDA by dioxastannolanes **8** (Figure 3) has been thoroughly investigated by Luchinat and Roelens [7]. Specifically, the authors reacted chiral 1,2-diols with achiral dialkyltin(IV) reagents to form the corresponding chiral dioxastannolanes. As these latter were known to the authors to strongly associate in apolar solvents, homo- and heterodimeric aggregates in diastereotopic relations were formed. In the condition of fast exchange of monomeric units among the dimers, two signals corresponding to the respective enantiomers were observed. This fast exchange condition was mediated through the formation of little amounts of higher-order aggregates. The achiral dialkyltin(IV) reagents served, therefore, as mediators of the dimerization. The authors found out that ^13^C NMR spectra were more suitable for the enantiomeric excess determination, as ^1^H NMR spectra were more complex.

**Figure 10:**
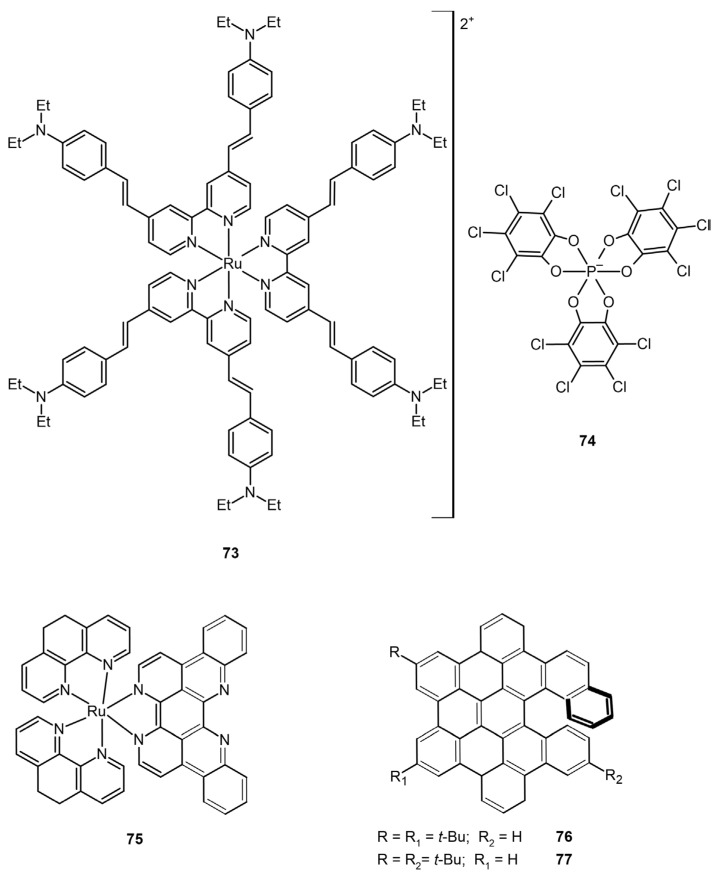
Ruthenium complexes and helicenes.

**Figure 11:**
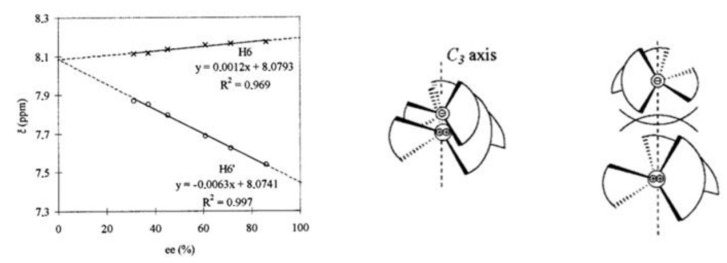
(**Left**) chemical shift variation measured for the ortho protons of the bipyridyl ring of **73,** dependent on the enantiomeric excess of **74**. (**Right**) representation of the homochiral and heterochiral interaction. Reprinted with permission from ref. [64].

Diastereoselective self-assembly occurs also in symmetric propeller-like molecules, which are of interest for their applications as chromophores for nonlinear optics. The complex given by the racemic ruthenium complex **73** and the racemic phosphate anion **74** (Figure 10) was synthesized and analyzed via ^1^H NMR spectroscopy [64]. The proton spectrum of the mixture, in which the presence of the two diastereomers given by the homochiral (ΛΛ/ΔΔ) and heterochiral (ΛΔ/ΔΛ) complexes was expected, was characterized instead by only one set of signals belonging to a single couple of enantiomers. Such results could be explained by considering either a dynamic phenomenon (one of the two couples converts into the other one) or the formation of one specific diastereomer. The spectra were then recorded at lower temperatures, but no splitting of signals occurred. The authors focused at this point on the analysis of enantiomerically enriched mixtures, where splitting of signals was observed. In particular, the ortho protons of the bipyridyl ring underwent a different variation in chemical shift, compared with the racemate, for the homo- and the heterocomplex, which were still linearly dependent on the enantiomeric excess of the anion **74** (Figure 11).

The different slope observed for the two lines pointed out a preference towards the formation of a specific complex; the homochiral one in this case. The proton spectrum of the racemate, thus, was explained by considering a fast-exchange equilibrium between homo- and heterochiral complexes, and the shift observed when lowering the temperature was ascribed to the shift of the equilibrium towards the formation of the more stable homochiral complex. Such stability was mainly related to lower electrostatic interactions in the homochiral complex (Figure 11).

The self-aggregating behavior of the ruthenium complexes **9** (Figure 3) and **75** (Figure 10) was investigated in CD_3_CN solutions to favor the occurrence of π-stacking interactions that are at the basis of the self-assembly [23]. The proton spectra recorded by changing the temperature and the concentration of the solutions highlighted a variation in chemical shift mainly for the eilatin protons. This trend pointed out not only the occurrence of an equilibrium between monomeric and dimeric species in solution but also the molecular moiety more involved in the supramolecular aggregation. The smaller ligands, on the other hand, were responsible for a well-defined arrangement of the dimers and, most importantly, their steric hindrance could play a primary role in favoring the formation of homochiral dimers over heterochiral ones or vice versa. The dilution method was exploited for calculating the homodimerization and the mean constant for substrate **9**, from which the heterodimerization constant was extrapolated according to Equation (63) (see Section 2.1). This approach revealed that K_d(hetero)_ was much higher than K_d(homo)_ and, therefore, that the formation of the heterochiral dimers was more favored in the racemic sample. A few years later, Kol extended the study to other chiral Ru complexes with eilatin, isoeilatin, phenazine, and dibenzoeilatin [65].

Helicenes also constitute an interesting class of compounds thanks to their electronic, magnetic, and spin properties, and the synthesis of laterally π-extended carbo[n]helicenes is an important target in organic chemistry. Derivatives **76** and **77** (Figure 10) have been synthetized starting from suitable triynes that were converted into other helicene intermediates via formation of a Rh complex, and a subsequent Scholl’s reaction yielded compounds **76** and **77** [66]. The helicene derivatives were analyzed in CDCl_3_. The ^1^H NMR analysis of enantiopure and racemic samples at different concentrations of both **76** and **77** resulted in a chemical shift dependance on the total concentration. Specifically, enantiopure samples resonated at lower frequency than racemic ones at the same concentration. According to the authors, this was an indication that enantiopure samples tended to self-aggregate more than racemic ones, hence, a stronger affinity toward homo- against heteroaggregation was pointed out. Furthermore, the differences between the ^1^H NMR spectra of nearly enantiopure forms and racemic samples at the same concentration served as an anticipation of the occurrence of SIDA. Indeed, scalemic mixtures of **76** (total concentration of 10 mM) and **77** (total concentration of 7.5 mM) yielded ^1^H NMR spectra in which some signals were split, and the integrated areas corresponded to the enantiomeric composition. The authors agreed that only a fast-exchange condition of enantiomeric monomers among the homo- and heteroaggregates could afford such splitting, namely the necessary condition for SIDA to occur. The proton spectra of scalemates showed splitting of signals belonging to the aromatic protons, in agreement with the occurrence of SIDA via π–π stacking, already observed between *P* and *M* enantiomers in the solid state [66]. 

## 4. Final Remarks

The theoretical treatments for describing the SIDA phenomenon can be classified in two main categories: the ones that consider only the presence of dimeric species [3,4,7] and those that also consider the presence of monomers [19,20,24]. The choice between one of the two types of theoretical approaches depends on whether the presence of monomeric species can be neglected. It is important to keep in mind that the values of the dimerization constants determined via one of the methods described can be used to calculate the fraction of the monomeric species present in solution. If this value is negligible with respect to the total concentration, it is reasonable then to consider the presence of dimers only in solution. In this case, the sole equilibrium established in solution is the interchange between the dimeric species (Equilibrium (3)).

Another important feature that arises is that a thermodynamic preference toward formation of homodimeric or heterodimeric species is not a mandatory requirement for SIDA to occur in enantiomerically enriched mixtures.

As far as the practical aspects of the phenomenon are concerned, it is possible to consider SIDA as both an opportunity and an issue in the analysis of chiral molecules that are likely to self-aggregate. As stated by Klika and coworkers [50], a peak arising from a minor enantiomer can be misrepresented as an impurity; therefore, it is important to consider the possibility that the substrate under investigation might be involved in the formation of diastereomeric adducts in solution so that the chemical purity can be correctly estimated. In the case of chiral molecules, the comparison between the spectra of enantiomerically pure mixtures and racemic ones at the same concentration can serve as a rapid test for assessing the occurrence of a particular case of aggregation, the so-called SIDA, given that in this case a difference in the chemical shift will be detected between the spectra of the two samples.

At the same time, SIDA presents the great advantage of allowing the determination of the enantiomeric excess of scalemates without using any chiral auxiliary, in addition to the fact that the nonequivalence becomes dependent from the enantiomeric composition, thus assuming particularly high values in cases of very high enantiomeric excesses. This aspect determines an improvement of the accuracy in the quantitative analysis, of particular relevance for the pharmaceutical market.

Additionally, the understanding of self-discrimination phenomena can facilitate the optimization of reaction conditions [39] and give clues to the origin of the homochirality that we observe today [67].

## Figures and Tables

**Table 1:** Symbols and definitions adopted.

Symbol	Definition	δ	Definition
E/E′	enantiomer/opposite enantiomer	δ_m_	monomer chemical shift
EE/E′E′	homodimers	δ_homo_	homodimer chemical shift
EE′	heterodimer	δ_hetero_	heterodimer chemical shift
D/D′	diastereomer pairs	δ_D_/δ_D′_	diastereomers chemical shift

## Data Availability

No new data was created or analyzed in this study.

## References

[1] Ouryupin A.B., Kadyko M.I., Petrovskii P.V., Fedin E.I. (1994). Chiral discrimination in nonracemic mixtures of methanephosphonic acid, *N*,*N*′-bis(1-phenylethyl)diamides. Chirality.

[2] Williams T., Pitcher R.G., Bommer P., Gutzwiller J., Uskokovic M. (1969). Diastereomeric solute-solute interactions of enantiomers in achiral solvents. Nonequivalence of the nuclear magnetic resonance spectra of racemic and optically active dihydroquinine. J. Am. Chem. Soc..

[3] Kabachnik M.I., Mastryukova T.A., Fedin E.I., Vaisberg M.S., Morozov L.L., Petrovsky P.V., Shipov A.E. (1976). An NMR study of optical isomers in solution. Tetrahedron.

[4] Kabachnik M.I., Mastryukova T.A., Fedin E.I., Vaisberg M.S., Morozov L.L., Petrovskii P.V., Shipov A.E. (1978). Optical Isomers in Solution Investigated by Nuclear Magnetic Resonance. Russ. Chem. Rev..

[5] Harger M.J.P. (1976). Nuclear magnetic resonance non-equivalence of the enantiomers in optically active samples of phosphinic amides. J. Chem. Soc. Chem. Commun..

[6] Dobashi A., Saito N., Motoyama Y., Hara S. (1986). Self-induced nonequivalence in the association of d- and l-amino acid derivatives. J. Am. Chem. Soc..

[7] Luchinat C., Roelens S. (1986). Enantiomeric purity determination of 1,2-diols through NMR spectroscopy without chiral auxiliaries. J. Am. Chem. Soc..

[8] Giordano C., Restelli A., Villa M., Annunziata R. (1991). A case of self-induced anisochrony in the proton NMR spectra of 1,5-benzothiazepines. J. Org. Chem..

[9] Szántay C., Demeter Á., Szántay C. (2015). Chapter 14—Self-Induced Recognition of Enantiomers (SIRE) in NMR Spectroscopy. Anthropic Awareness.

[10] Martens J., Bhushan R. (2014). Purification of Enantiomeric Mixtures in Enantioselective Synthesis: Overlooked Errors and Scientific Basis of Separation in Achiral Environment. Helv. Chim. Acta.

[11] Soloshonok V.A. (2006). Remarkable Amplification of the Self-Disproportionation of Enantiomers on Achiral-Phase Chromatography Columns. Angew. Chem. Int. Ed..

[12] Han J., Wzorek A., Kwiatkowska M., Soloshonok V.A., Klika K.D. (2019). The self-disproportionation of enantiomers (SDE) of amino acids and their derivatives. Amino Acids.

[13] Balzano F., Uccello Barretta G., Aiello F., Polavarapu P.L. (2018). Chapter 9—Chiral Analysis by NMR Spectroscopy: Chiral Solvating Agents. Chiral Analysis.

[14] Soloshonok V.A., Wzorek A., Klika K.D. (2017). A question of policy: Should tests for the self-disproportionation of enantiomers (SDE) be mandatory for reports involving scalemates?. Tetrahedron Asymmetry.

[15] Horeau A., Guetté J.P. (1974). Interactions diastereoisomeres d’antipodes en phase liquide. Tetrahedron.

[16] Kabachnik M.I. (1977). Associate diasteromerism of organophosphorus compounds. Phosphorus Sulfur Relat. Elem..

[17] Cung M.T., Marraud M., Neel J. (1977). Etude experimentale de L’auto-association des molécules modèles dipeptidiques. III. Influence de la dimerisation stéréosélective sur les spectres de résonance magnétique protonique. Biopolymers.

[18] Avignon M., Huong P.V., Lascombe J., Marraud M., Neel J. (1969). Etude, par spectroscopie infra-rouge, de la conformation de quelques composés peptidiques modèles. Biopolymers.

[19] Cung M.T., Marraud M., Neel J., Aubry A. (1978). Experimental study on aggregation of model dipeptide molecules. V. Stereoselective association of leucine dipeptides. Biopolymers.

[20] Nakao Y., Sugeta H., Kyogoku Y. (1985). Intermolecular Hydrogen Bonding of Enantiomers of Pantolactone Studied by Infrared and ^1^H-NMR Spectroscopy. Bull. Chem. Soc. Jpn..

[21] Sugeta H. (1981). Spectrophotometric Determination of Formation Constants and Estimation of Molar Absorption Spectra of Individual Components in Chemical Equilibria. Infrared Study of Intermolecular Hydrogen Bonding of 2-Aminopyridine. Bull. Chem. Soc. Jpn..

[22] Fedin E.I., Davankov V.A. (1995). NMR investigations of the enantiomeric excess effects in solutions with weak intermolecular association. Chirality.

[23] Gut D., Rudi A., Kopilov J., Goldberg I., Kol M. (2002). Pairing of Propellers:  Dimerization of Octahedral Ruthenium(II) and Osmium(II) Complexes of Eilatin via π−π Stacking Featuring Heterochiral Recognition. J. Am. Chem. Soc..

[24] Szakács Z., Sánta Z., Lomoschitz A., Szántay C. (2018). Self-induced recognition of enantiomers (SIRE) and its application in chiral NMR analysis. TrAC Trends Anal. Chem..

[25] Ouryupin A.B., Kadyko M.I., Petrovskii P.V., Fedin E.I., Okruszek A., Kinas R., Stec W.J. (1995). Enantiomeric 2-anilino-2-oxo-1,3,2-oxazaphosphorinanes: Synthesis and NMR-investigation of their non-racemic mixtures. Tetrahedron Asymmetry.

[26] Han J., Fustero S., Moriwaki H., Wzorek A., Soloshonok V.A., Klika K.D., Szabó K., Selander N. (2021). The Self-Disproportionation of Enantiomers (SDE): Fluorine as an SDE-Phoric Substituent. Organofluorine Chemistry.

[27] Klika K.D., Budovská M., Kutschy P. (2010). Enantiodifferentiation of phytoalexin spirobrassinin derivatives using the chiral solvating agent (*R*)-(+)-1,1′-bi-2-naphthol in conjunction with molecular modeling. Tetrahedron Asymmetry.

[28] Nieminen V., Murzin D.Y., Klika K.D. (2009). NMR and molecular modeling of the dimeric self-association of the enantiomers of 1,1′-bi-2-naphthol and 1-phenyl-2,2,2-trifluoroethanol in the solution state and their relevance to enantiomer self-disproportionation on achiral-phase chromatography (ESDAC). Org. Biomol. Chem..

[29] Horman I., Dreux B. (1984). Estimation of Dimerisation Constants from Complexatin-Induced Displacements of ^1^H-NMR Chemical Shifts: Dimerisation of Caffeine. Helv. Chim. Acta.

[30] Chen J.S., Shirts R.B. (1985). Iterative determination of the NMR monomer shift and dimerization constant in a self-associating system. J. Phy. Chem..

[31] Jenn-shing C., Rosenberger F. (1990). Accurate nmr data evaluation for monomer shift, dimer shift and dimerization constant in a self-associating system. Tetrahedron Lett..

[32] Brynn Hibbert D., Thordarson P. (2016). The death of the Job plot, transparency, open science and online tools, uncertainty estimation methods and other developments in supramolecular chemistry data analysis. Chem. Commun..

[33] Nelder J.A., Mead R. (1965). A Simplex Method for Function Minimization. Comput. J..

[34] Kabachnik M.I., Mastryukova T.A., Fedin É.I., Shipov É.A., Vaisberg M.S., Petrovskii P.V., Morozov L.L. (1975). Study of diastereomeric anisochronism of some O-ethyl methyldithiophosphonates that contain a second chiral center in the thiol group. Bull. Acad. Sci. USSR Div. Chem. Sci..

[35] Kabachnik M.I., Fedin É.I., Morozov L.L., Vaisberg M.S., Petrovskii P.V., Shipov A.É., Mastryukova T.A. (1976). Diastereomeric anisochronism in rapid interaggregate exchange. Bull. Acad. Sci. USSR Div. Chem. Sci..

[36] Harger M.J.P. (1977). Proton magnetic resonance non-equivalence of the enantiomers of alkylphenylphosphinic amides. J. Chem. Soc. Perkin Trans. 2.

[37] Harger M.J.P. (1978). Chemical shift non-equivalence of enantiomers in the proton magnetic resonance spectra of partly resolved phosphinothioic acids. J. Chem. Soc. Perkin Trans. 2.

[38] Mikołajczyk M., Żurawiński R., Kiełbasiński P., Wieczorek M.W., Błaszczyk J., Majzner W.R. (1997). Total Synthesis of Racemic and Optically Active Sarkomycin. Synthesis.

[39] Dašková V., Padín D., Feringa B.L. (2022). Turning Enantiomeric Relationships into Diastereomeric Ones: Self-Resolving α-Ureidophosphonates and Their Organocatalytic Enantioselective Synthesis. J. Am. Chem. Soc..

[40] Cung Manh T., Marraud M., Neel J. (1975). Estimation of the optical purity of chiral peptide derivatives by proton NMR spectroscopy. C. R. Hebd. Seances Acad. Sci. Ser. C.

[41] Cung M.T., Marraud M., Neel J. (1976). Étude expérimentale de l’auto-association des molécules modèles dipeptidiques. II. Association stéréosélective des molécules énantiomères. Biopolymers.

[42] Ghosh S.K. (1999). Chiral recognition in dipeptides containing 1-aminocyclopropane carboxylic acid or α-aminoisobutyric acid: NMR studies in solution. J. Peptide Res..

[43] Kuberski B., Szumna A. (2009). A self-assembled chiral capsule with polar interior. Chem. Commun..

[44] Storch G., Haas M., Trapp O. (2017). Attracting Enantiomers: Chiral Analytes That Are Simultaneously Shift Reagents Allow Rapid Screening of Enantiomeric Ratios by NMR Spectroscopy. Chem. Eur. J..

[45] Jursic B.S., Goldberg S.I. (1992). Enantiomer discrimination arising from solute-solute interactions in partially resolved chloroform solutions of chiral carboxamides. J. Org. Chem..

[46] Wagger J., Grdadolnik S.G., Grošelj U., Meden A., Stanovnik B., Svete J. (2007). Chiral solvating properties of (*S*)-1-benzyl-6-methylpiperazine-2,5-dione. Tetrahedron Asymmetry.

[47] Huang S.-H., Bai Z.-W., Feng J.-W. (2009). Chiral self-discrimination of the enantiomers of α-phenylethylamine derivatives in proton NMR. Magn. Reson. Chem..

[48] Safont-Sempere M.M., Osswald P., Radacki K., Würthner F. (2010). Chiral Self-Recognition and Self-Discrimination of Strapped Perylene Bisimides by π-Stacking Dimerization. Chem. Eur. J..

[49] Safont-Sempere M.M., Osswald P., Stolte M., Grüne M., Renz M., Kaupp M., Radacki K., Braunschweig H., Würthner F. (2011). Impact of Molecular Flexibility on Binding Strength and Self-Sorting of Chiral π-Surfaces. J. Am. Chem. Soc..

[50] Baumann A., Wzorek A., Soloshonok V.A., Klika K.D., Miller A.K. (2020). Potentially Mistaking Enantiomers for Different Compounds Due to the Self-Induced Diastereomeric Anisochronism (SIDA) Phenomenon. Symmetry.

[51] Arnold W., Daly J.J., Imhof R., Kyburz E. (1983). An efficient resolution of 3-PPP and assignment of the absolute configuration. Tetrahedron Lett..

[52] Sonstrom R.E., Neill J.L., Mikhonin A.V., Doetzer R., Pate B.H. (2022). Chiral analysis of pantolactone with molecular rotational resonance spectroscopy. Chirality.

[53] Hui R.A.H.F., Salamone S., Williams T.H. (1991). Self-induced nonequivalence in the ^1^H-NMR spectra of the (+)- and (−)-isomers of a cannabinoid ketone intermediate. Pharmacol. Biochem. Behav..

[54] Srinivasan K., Preedy V.R. (2014). Chapter 42—Polyphenols in Vision and Eye Health. Handbook of Nutrition, Diet and the Eye.

[55] Tait A., Colorni E., Di Bella M. (1997). Stereospecific synthesis of 2-[(2H-1,2,4-benzothiadiazine-1,1-dioxide-3-yl)thio]propanoic acids: Enantiomeric excess evaluation by ^1^H NMR. Tetrahedron Asymmetry.

[56] Hentschel P., Holtin K., Steinhauser L., Albert K. (2012). Monitoring the On-line Titration of Enantiomeric Omeprazole Employing Continuous-flow Capillary Microcoil ^1^H NMR Spectroscopy. Chirality.

[57] Kwiatkowska M., Wzorek A., Kolbus A., Urbaniak M., Han J., Soloshonok V.A., Klika K.D. (2021). Flurbiprofen: A Study of the Behavior of the Scalemate by Chromatography, Sublimation, and NMR. Symmetry.

[58] Klika K.D., Budovská M., Kutschy P. (2010). NMR spectral enantioresolution of spirobrassinin and 1-methoxyspirobrassinin enantiomers using (*S*)-(−)-ethyl lactate and modeling of spirobrassinin self-association for rationalization of its self-induced diastereomeric anisochromism (SIDA) and enantiomer self-disproportionation on achiral-phase chromatography (ESDAC) phenomena. J. Fluor. Chem..

[59] Budovská M., Tischlerová V., Mojžiš J., Harvanová M., Kozlov O., Gondová T., Tomášková N. (2017). 2′-Aminoanalogues of the cruciferous phytoalexins spirobrassinin, 1-methoxyspirobrassinin and 1-methoxyspirobrassinol methyl ether: Synthesis and anticancer properties. Tetrahedron.

[60] Xu Z., Wang Q., Zhu J. (2015). Total Syntheses of (−)-Mersicarpine, (−)-Scholarisine G, (+)-Melodinine E, (−)-Leuconoxine, (−)-Leuconolam, (−)-Leuconodine A, (+)-Leuconodine F, and (−)-Leuconodine C: Self-Induced Diastereomeric Anisochronism (SIDA) Phenomenon for Scholarisine G and Leuconodines A and C. J. Am. Chem. Soc..

[61] Charpentier M., Hans M., Jauch J. (2013). Enantioselective Synthesis of Myrtucommulone A. Eur. J. Org. Chem..

[62] Ajisaka K., Kainosho M. (1975). Diastereomeric interaction of partially resolved amines facilitated by lanthanide chelates. Evidence for dynamic equilibrium between seven-coordinate and eight-coordinate alkylamine-lanthanide chelate adducts. J. Am. Chem. Soc..

[63] Reuben J. (1980). Aqueous lanthanide shift reagents. 8. Chiral interactions and stereochemical assignments of chemically and isotopically chiral ligands. J. Am. Chem. Soc..

[64] Maury O., Lacour J., Le Bozec H. (2001). Diastereoselective Homochiral Self-Assembly Between Anions and Cation in Solution. Eur. J. Inorg. Chem..

[65] Bergman S.D., Kol M. (2005). π-Stacking Induced NMR Spectrum Splitting in Enantiomerically Enriched Ru(II) Complexes:  Evaluation of Enantiomeric Excess. Inorg. Chem..

[66] Morita F., Nogami J., Araujo Dias A.J., Kinoshita S., Nagashima Y., Tanaka K. (2022). Regiodivergent Synthesis and π-Stacking-Induced Chiral Self-Recognition of Hexabenzocoronene-Based [6]Helicenes. Eur. J. Org. Chem..

[67] Fedin E.I. NMR of Chiral Molecules. Proceedings of the 20th Congress AMPERE.

